# Artificial intelligence in clinical trial participant recruitment and retention: A scoping review and meta-analysis

**DOI:** 10.1017/cts.2026.10743

**Published:** 2026-04-23

**Authors:** Ziran Yin, Yun-Chung Liu, Jonathan Chong Kai Liew, Rui Yang, Stephanie Hendren, Elisa Ma, Zhaomei Geng, Jiahan Wang, Henry Foote, Christopher Lindsell, Chuan Hong

**Affiliations:** 1 Biostatistics and Bioinformatics, Duke University, NC, USA; 2 Duke University, NC, USA; 3 School of Chemistry, Chemical Engineering and Biotechnology, Nanyang Technological University, Singapore, Singapore; 4 Department of Biomedical Informatics, National University of Singapore, Singapore, Singapore; 5 Herbert Wertheim School of Public Health and Human Longevity Science, University of California San Diego, CA, USA; 6 University of Wisconsin-Madison, WI, USA; 7 Duke Clinical Research Institute, Duke University, NC, USA

**Keywords:** Clinical trials, artificial intelligence, recruitment, retention, meta analysis

## Abstract

Recruitment and retention challenges continue to hinder the success of clinical trials. Artificial intelligence (AI) has emerged as a promising means to optimize various clinical trial processes; however, its impact specifically on recruitment and retention has not been comprehensively evaluated. This scoping review utilized the Joanna Briggs Institute framework and adhered to PRISMA-ScR guidelines, systematically searching literature published between January 2018 and June 2024 across multiple databases. Of the 21,573 records screened, 121 studies were included. A meta-analysis was conducted to quantitatively assess the performance of AI-driven tools. AI applications for patient screening demonstrated strong performance, achieving a pooled sensitivity of 0.91 (95% CI: 0.84–0.95) and an area under the curve (AUC) of 0.79 (95% CI: 0.72–0.85). AI tools employed for eligibility identification and classification also exhibited strong outcomes, with pooled sensitivities of 0.80 (95% CI: 0.76–0.84) and 0.92 (95% CI: 0.84–0.96), respectively, and precisions of 0.84 (95% CI: 0.80–0.88) and 0.91 (95% CI: 0.85–0.95). AI tools aimed at identifying patient cohorts showed moderate effectiveness (pooled sensitivity: 0.70 [95% CI: 0.52–0.84]; AUC: 0.74 [95% CI: 0.61–0.84]). Overall, AI presents significant potential for enhancing clinical trial recruitment and retention, with effectiveness varying across specific applications. These findings underscore AI’s valuable role in improving trial efficiency and data quality.

## Introduction

Clinical trials are critical to advance medical treatments from the laboratory to the bedside. However, this process is lengthy and costly, costing up to 10 to 15 years and as much as $2 billion [1–3]. Nearly 40% of trial sites face under-recruitment, and delays in recruitment can extend the study duration by 1 to 6 months, making trials less effective and efficient [2,4]. In addition, traditional recruitment methods are not only labor-intensive but also often biased; studies have shown that older adults, women, and minority ethnic groups are frequently underrepresented, undermining the external validity of trial results [5–9]. These challenges can delay new therapies, incur economic losses, and raise ethical concerns about equitable trial access.

Advances in artificial intelligence (AI) have begun to transform the healthcare industry in recent years. AI tools encompass various categories, including machine learning (supervised, unsupervised, reinforcement), robotic, and rule-based approaches [10,11]. Machine learning-based AI excels with large, unstructured data and complex tasks, while rule-based and robotic AIs perform better with structured data and simple tasks [12–14]. From early predictive modeling to complex algorithms involving natural language processing (NLP), large-scale language models, and interactive chatbots, developments in AI have opened new avenues for addressing long-standing challenges in clinical trials. The application of AI in diagnostic imaging, dose management, and clinical decision-making has yielded promising results [15–17].

However, integrating AI into clinical trial program workflows remained a challenge. Issues of interoperability, clinician trust, adoption, and the risk of embedding or exacerbating bias through AI algorithms remained serious concerns. These challenges are critical given the need for diverse and inclusive patient populations in clinical trials [18–21]. While AI has been credited for its ability to optimize the clinical trial process [22–25], relatively few studies have explored the role of AI in improving early-stage trial efficiency, particularly through improved recruitment and retention.

We conducted a comprehensive scoping review to evaluate the role, effectiveness, and limitations of AI-powered tools in clinical trial recruitment and retention. By synthesizing existing evidence, we highlight both the promise and challenges of current applications, aiming to inform future strategies for effective and equitable implementation.

## Methods

We conducted a comprehensive scoping review to investigate the use of AI technologies in enhancing the recruitment and retention process in clinical trials. To accomplish this, this review followed the methodological framework outlined by the Joanna Briggs Institute (JBI). It was reported using the Preferred Reporting Items for Systematic Reviews of Scoping Reviews (PRISMA-ScR) guidelines to ensure transparency and compliance with reporting standards [26–28]. The paper screening process was described by the PRISMA-ScR flowchart. The included papers were carefully categorized. The performance of the AI models was then analyzed using a comprehensive meta-analysis. Details of the methodological framework for the beginning phase can be found in Supplementary Material 1 (*Appendix A: Extra Details of Methods of the Appendix.additional content*).

### Developing the search strategy

A comprehensive search strategy was developed for each database with the help of an experienced librarian to identify relevant studies. The search strategy combined keywords and database-specific controlled vocabulary using “OR” and filtered papers on AI and trial participant terms/keywords using “AND.” Searches were conducted in multiple databases, including comprehensive searches in Medline (via Ovid), Cochrane Library, Embase (Elsevier), CINAHL Complete (EBSCOhost), and Scopus (Elsevier) using controlled vocabulary and keywords. Only publications written in English were included to ensure feasibility, and comments, editorials, letters, and conference abstracts were excluded from the search. The literature search was limited to publications from January 1, 2018, to June 28, 2024, coinciding with the emergence of new AI tools and techniques. The search strategy was adapted to match different formats from different databases. An experienced librarian assisted throughout the construction, conversion, and implementation of the search strategy, ensuring the robustness of the search and the comprehensive identification of relevant studies. Details of our search strategy can be found in Supplementary Material 1 (*Appendix B: Search Strategy of the Appendix.additional content*).

### Paper screening and source of evidence selection

The paper screening process followed a structured approach [26,29] to ensure comprehensive and unbiased inclusion of relevant evidence. Titles and abstracts were initially screened using Covidence, followed by full-text review, with all studies assessed by at least two independent reviewers. Discrepancies were resolved through group discussion with an additional reviewer. Reviewers (YC, ZR, JCKL, YR, EM, ZG) received training and completed a 100-title pilot to ensure consistency. Dual independent screening was applied to all records, with conflicts resolved by third reviewers (HF, CH). Screening results are detailed in the results section and illustrated in the PRISMA-ScR flowchart.

Large language model-based assistance (ChatGPT, OpenAI) was used to improve the efficiency of title and abstract screening. We applied the predefined inclusion criteria to generate a relevance score (0–100). These scores were used to prioritize the order of human review (≥58 indicating likely to meet, 40–58 ambiguous, <40 unlikely to meet). All results were subsequently manually checked and verified by reviewers (YC, ZR, JCKL, YR, EM, ZG). Further methodological details are provided in Supplementary Material 1 (*Appendix A: Extra Details of Methods of the Appendix.additional content*). The detailed description of our AI-assisted methodological construction is discussed in Liu *et al* [30].

### Charting the evidence and data extraction

We extracted key information from each included study, including demographic features, AI tool details, and AI model performance. Study characteristics included study purpose, publication date, duration, country of author affiliation, cohort description, inclusion and exclusion criteria, and dataset sources. AI tool implementation was assessed across four aspects: (1) tool timing (retrospective analysis vs. real-time alert system); (2) tool implementation (theoretical framework vs. real-world clinical setting); (3) type of tool (recruitment AI vs. retention AI); and (4) type of AI technology (e.g., electronic health record (EHR)-based models, chatbot-based recruitment, NLP for eligibility screening, or machine learning-based patient stratification). AI model performance was documented using clinical outcome metrics (e.g., time saved, percentage of participants retained, trial efficiency) and standard metrics: precision, recall, positive predictive value (PPV), negative predictive value (NPV), area under the receiver operating characteristic curve (AUC-ROC), and F1-score.

### Paper categorization by recruitment and retention workflow

To enhance interpretability and facilitate subsequent meta-analysis, we categorized each study according to the specific stage of the clinical trial recruitment and retention workflow that the AI tool aimed to enhance. The included studies were grouped into 10 categories. The first category includes AI tools that extract, structure, and interpret eligibility criteria. The second focuses on classifying free-text eligibility into structured formats. The third involves identifying potential patient cohorts, followed by tools for direct patient screening that assess trial eligibility. Next, AI tools focused on patient-trial matching systematically pair patient profiles with suitable clinical trials or arms. Upon completion of the recruitment stage, the retention phase begins with AI tools that enhance patient adherence during retention. Two additional categories include AI tools for evaluating and optimizing past trial eligibility rules and AI-driven digital platforms that enhance trial processes or deliver clinical information to stakeholders. Beyond the categorization, studies were also classified by (1) timing, (2) implementation, and (3) tool type, as previously described.

### Meta-analysis

We conducted a meta-analysis stratified by clinical trial categories to quantitatively assess the AI model’s performance at different stages of the recruitment and retention workflow. Each meta-analysis used performance metrics, including sensitivity, specificity, precision, accuracy, F1 score, and AUC. A random-effects model was employed to account for heterogeneity among studies, and weighted averages were calculated for pooled performance estimates. Forest plots were generated for each model’s performance to visualize individual study contributions and overall effect sizes. The between-study heterogeneity was quantified using k, *τ* ^2^, and *I* ^2^ for each task–metric stratum, and results were interpreted. The full stratum-level table could be referred to Supplementary Material 2 (*Appendix Table 1. heterogeneity assessment*).

Detailed code and data tables are provided in the accompanying GitHub repository: https://github.com/yinz33/Artificial-Intelligence-in-Clinical-Trial-Participant-Recruitment-and-Retention.git

## Results

### Applications of artificial intelligence across the clinical trial workflow

#### Study identification and screening summary

**Figure 1.**
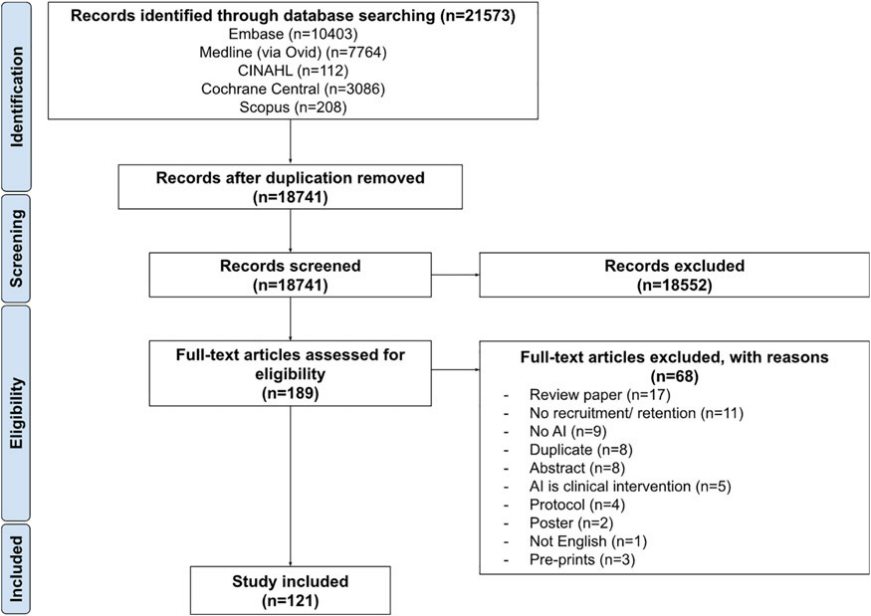
PRISMA-ScR flowchart for the scoping review process. Out of 189 full-text articles that passed the title and abstract screening, 68 were excluded primarily because they were review papers, lacked AI application, or did not focus on recruitment or retention.

21,573 records were initially identified through our database searches (Embase, Medline [via Ovid], CINAHL, CENTRAL, and Scopus). After removing duplicates, 18,741 unique records underwent title and abstract screening. The low inclusion rate of 1.01% reflects our deliberate use of broad search terms to ensure comprehensive coverage of this emerging field, combined with rigorous inclusion criteria requiring demonstrated AI implementation rather than theoretical discussions. Many retrieved studies addressed AI in clinical settings generally, but relatively few specifically focused on recruitment and retention processes within clinical trial workflows. This stringent approach ensures our findings represent the current evidence base of practically implemented AI solutions in clinical trials. 18,552 articles were excluded during title and abstract screening. 194 disagreements (1.04%) from independent double-screenings were reconciled on multiple occasions. Table 1 outlines the key features of studies that utilize AI tools to screen and recruit eligible patients. Details of addressing disagreement can be referred to Supplementary Material 3 (*Appendix Table 2. screening and resolution*). Subsequently, 189 articles were assessed in full text, and 68 were excluded. Ultimately, 121 articles were included in this review (Figure 1).

**Table 1.** Brief summary of included studies

Citation	Recruitment/retention	Cohort description	AI tech description
Haddad et al., 2021 [63]	Recruitment	318 patients with breast cancer were treated in the medical oncology clinic at Mayo Clinic in Rochester, Minnesota, between May and July of 2017, with at least one unstructured health record note for processing by the CDSS (clinical decision support system).	Study Period: May–July 2017 Tool: Natural Language Processing Focus: Identifies cancer stage, subtype, genetic markers, prior therapy, surgical history, and therapy-related characteristics to support patient characterization.
Chen et al., 2019 [118]	Recruitment	100,134 clinical trials from ClinicalTrials.gov to address gender inclusivity in eligibility criteria, particularly for the transgender population, with manually annotated 134 transgender-recruiting trials as the gold standard of evaluation.	Study period: not specified Tool: Rule-based organizer Focus: Automate information extraction and summarization to improve gender requirement specifications in clinical trial.
Hassan et al., 2023 [115]	Recruitment	Patients with acute SDH, limited life expectancy, severe grading scores, anatomical risks, or contraindications to treatment were excluded. Four patients were recruited using AI, while five were recruited without AI.	Study period: October 6, 2021–August 18, 2022 Tool: An AI-powered software platform Focus: Automatically identified SDH in non-contrast computed tomography (NCHCT) scans, measuring volume, maximum thickness, and midline shift. The results were sent to the stroke care team via a mobile app, and notifications indicated whether patients met predefined eligibility criteria.
Beck et al., 2020 [119]	Recruitment	Patients in both groups were predominantly female, aged between 25 and 98 years, and most had hormone receptor-positive, HER2-negative breast cancer.	Study Period: not specified Tool: NLP to process unstructured information Focus: To reduce screening time, incorporate structured data (such as lab results, sex, cancer diagnosis, and age) and unstructured sources (like recent medical notes).
Zeng et al., 2021 [44]	Recruitment	The study used 38,341 clinical trial eligibility criteria texts from a dataset manually annotated into 44 categories based on CHIP 2019 Task 3. The dataset was divided into a training set (22,962 texts), a validation set (7682 texts), and a test set (7697 texts).	Study Period: 2019, but no specification of start and end dates Tool: Integrated machine-learning algorithm with BERT, XLNet, RoBERTa, and ERNIE for text classification, with LightGBM used for final predictions Focus: Process unstructured eligibility criteria text, extract key features, and apply the local loss to handle class imbalance.
Kirshner et al., 2021 [120]	Recruitment	66,532 patients with solid tumors from Flatiron Health’s nationwide oncology database. The patients were identified using specific medical codes (International Classification of Diseases codes) and were confirmed by manual review.	Study Period: January 1, 2011 to June 30, 2019 Tool: NLP tool Focus: Automatically classify metastatic status from unstructured EHR data and categorize patients into five metastatic likelihood groups.
Kaskovich et al., 2023 [67]	Recruitment	The study utilized 216 Phase I–III leukemia trial protocols from ClinicalTrials.gov (1987–2018) as training data, including both completed and ongoing trials. Eligibility criteria were presented in varying formats, from simple bulleted lists (14%) to more complex structures with nested sub-bullets and category headers (43%).	Study Period: not specified Tool: NLP Focus: Extract inclusion and exclusion criteria from free-text clinical trial protocols and incorporate patient-specific demographic and clinical data to generate a ranked list of clinical trials based on the likelihood of patient eligibility.
Tun et al., 2023 [41]	Recruitment	The cohort was sourced from de-identified electronic medical records (EMRs) across multiple clinics, including data from 40,000 patients, including 109 individuals already known to meet the clinical trial inclusion/exclusion criteria.	Study Period: Tool: Integrated patient selection model with rule-based algorithms and pre-trained NLP biomedical text embedding model Focus: Process structured data (e.g., demographics, lab results) and unstructured clinician notes, managing inclusion/exclusion criteria that involve over 50 eligibility parameters.
Meystre et al., 2019 [42]	Recruitment	229 breast cancer patients treated at the Medical University of South Carolina (MUSC) Hollings Cancer Center between 2015 and 2017.	Study Period: 2015 to 2017 Tool: Observational Medical Outcomes Partnership (OMOP) Common Data Model, an NLP-based AI system Focus: Automatically extract eligibility criteria from unstructured clinical notes and map them to structured trial descriptions.
Murcia et al., 2024 [68]	Recruitment	Seven clinical trials were designed, with patients having cardiovascular diseases and posttraumatic stress disorder and a total cohort size of 3,956,913 participants.	Study Period: 2015 to 2017 Tool: NLP-powered clinical trial matching system Focus: Automatically detect a patient’s eligibility by mapping coded clinical trial eligibility criteria to the corresponding clinical information.
Jung et al., 2021 [31]	Recruitment	1058 clinical trial studies (Phase 1: 378, Phase 2: 664).	Study Period: Phase I: January 1, 2010 to January 1, 2011; Phase II: January 1, 2018 to January 1, 2019 Tool: NLP and text mining Focus: Enhance the efficiency of clinical trial-patient eligibility matching.
Oluoch et al., 2021 [94]	Retention	5901 adults and children (aged ≥18 months) receiving antiretroviral therapy at 20 HIV clinics in western Kenya.	Study Period: September 1, 2012 to January 31, 2014 Tool: EHR integrated model with a clinical decision support system (CDSS)-automated alert tool (with no machine-learning AI mentioned) Focus: Improve adherence to HIV clinical guidance, enhancing viral suppression.
Kehl et al., 2021 [121]	Recruitment	302,688 imaging reports for 16,780 patients who had genomic profiling performed through the Dana-Farber Cancer Institute PROFILE.	Study Period: not specified Tool: A deep neural network-based NLP model Focus: Analyze radiology reports and detect clinical inflection points designed for real-time screening of EHRs, enabling targeted trial recruitment at critical disease progression stages.
Chen et al., 2019 [122]	Recruitment	Patient records from the 2018 National NLP Clinical Challenge (n2c2) consist of 288 longitudinal patient records. Training set: 202 patients, Testing set: 86 patients.	Study Period: not specified Tool: Multi-level NLP system with a rule-based framework Focus: Automate cohort selection by analyzing unstructured clinical text.
Yang et al., 2018 [50]	Recruitment	264 study volunteers who passed the inclusion criteria were recruited for the SCIP-PA Trial (NCT01454830), middle-aged, overweight, or obese men and women undergoing polysomnography (PSG) for obstructive sleep apnea (OSA) diagnosis.	Study Period: not specified Tool: Multivariable Apnea Prediction (MAP) index Focus: Improve patient selection before diagnostic confirmation with the MAP index-screening tool for obstructive sleep apnea (OSA) risk.
Spasic et al., 2019 [123]	Recruitment	A total of 288 patients were included, with 202 used for training and 86 for testing. The study evaluated 13 eligibility criteria as part of the 2018 National NLP Clinical Challenges (n2c2).	Study Period: not specified Tool: A text-mining approach combining supervised machine learning (13 binary classifiers) and rule-based classification Focus: Analyze longitudinal patient records to determine if patients met predefined eligibility criteria for clinical trials.
Segura-Bedmar et al., 2019 [124]	Recruitment	The dataset from the 2018 n2c2 shared task (Track 1) focused on cohort selection for clinical trials. It comprised 311 patient records manually labeled by experts to determine whether each patient met any of the 13 predefined eligibility criteria.	Study Period: not specified Tool: NLP for cohort selection and various classical machine learning classifiers and a convolutional neural network (CNN) to automatically detect cases of anaphylaxis Focus: Enhance cohort selection to promote recruitment.
Xiong et al., 2019 [125]	Recruitment	288 patients were manually annotated with indicators of 13 predefined selection criteria.	Study Period: not specified Tool: Hierarchical neural network (Highway-LSTM or LSTM-Highway-LSTM) Focus: Automates eligible patient selection for clinical trials based on textual and/or structured EHR data.
Dai et al., 2020 [64]	Recruitment	288 patients, each with 2–5 records annotated with the binary tags “met” or “not met” for the 13 selection criteria.	Study Period: not specified Tool: Multiple algorithms, including MISVM, CitationKNN, miSVM, MIWrapper, Two-level classification, and simple MI Focus: Screening patients and identifying individuals who meet certain clinical or research eligibility benchmarks.
Fang et al., 2022 [32]	Recruitment	1010 COVID-19 clinical trials from ClinicalTrials.gov, information includes semantic complexities, negation scope, and temporal constraints.	Study Period: not specified Tool: Criteria2Query (C2Q) 2.0-entity recognition NLP, with a human in the loop Focus: Enable real-time user intervention for criteria selection and simplification, parsing error correction, and concept mapping.
Li et al., 2022 [126]	Recruitment	The Chia corpus dataset contains 1000 clinical trials from ClinicalTrials.go, 934 trials after cleaning removal.	Study Period: not specified Tool: Named entity recognition (NER) for unstructured text standardization and process (with pre-trained NLP: BioBERT, BlueBERT, PubMedBERT, and SciBERT) Focus: Extract accurate and meaningful information from the original clinical trials.
Li et al., 2021 [49]	Recruitment	1700 clinical trials from ClinicalTrials.gov; Three corpora were used: 470 in-house drug development study protocols (Covance), 230 Alzheimer’s disease trials (EliIE), and 1000 Phase IV interventional trials (Chia).	Study Period: not specified Tool: Named entity recognition (NER) for unstructured text standardization and process (with pre-trained NLP: BioBERT, BlueBERT, PubMedBERT, and SciBERT) Focus: Quantify and qualify the disagreement between humans and NLP in annotating clinical text, evaluating its performance for extracting accurate and meaningful information.
Chuan et al., 2021 [82]	Recruitment	The study gathered 209,441 eligibility criteria from 9762 clinical trials listed on the National Cancer Institute’s (NCI) website.	Study Period: not specified Tool: Sofia-a chatbot assistant based on deep learning and a multi-layer convolutional neural network (CNN) classifier Focus: Explain eligibility criteria, answer medical terminology questions, and streamline the verification process for patients in a user-friendly manner.
Park et al., 2024 [48]	Recruitment	20 clinical trials with free-text eligibility criteria from ClinicalTrials.gov covering various conditions, trial phases, and interventions were used to identify 518 clinical concepts.	Study Period: not specified Tool: C2Q 3.0-an integrated tool with OpenAI GPT-4, automates parsing, reasoning, and SQL queries Focus: Improve the accuracy of criteria parsing and query formulation from free-text eligibility criteria.
Yuan et al., 2019 [33]	Recruitment	The model evaluated 125 eligibility criteria from ClinicalTrials.gov across various disease domains and 52 user-entered criteria.	Study Period: not specified Tool: Criteria2Query system, a natural language interface (NLI) Focus: Translates free-text clinical trial eligibility criteria into structured, executable SQL queries for clinical databases.
Bompelli et al., 2020 [34]	Recruitment	Data was sourced from ClinicalTrials.gov, selecting 100 DS trials across two main disease categories: Behavioral & Mental Disorders and Nervous System Diseases (149 trials) and Nutritional and Metabolic Disorders (199 trials).	Study Period: not specified Tool: NLP models with deep learning application Focus: Examined dietary supplement (DS) clinical trials by automatically extracting entities and attributes from free-text eligibility criteria.
Jordan et al., 2021 [107]	Recruitment	29 gastrointestinal (GI) cancer patients across the United States, with support from patient associations and social media outreach.	Study Period: not specified Tool: NLP Focus: Restructure and standardize trial information, providing patient-friendly trial summaries in English and Spanish, enabling automated trial matching.
JuanRamon et al., 2024 [66]	Recruitment	The algorithm is validated on a dataset of 350 patients, with the data split preserving the same ratio of FGFR + vs. FGFR- patients and the proportion of samples from each cohort.	Study Period: not specified Tool: deep-learning algorithm Focus: Using >3000 H&E-stained whole slide images to train the model and improve diagnostic or prognostic accuracy for urothelial carcinoma.
Widera et al., 2023 [53]	Recruitment	Five European clinical centers utilized data from multiple cohorts with 3500 patients.	Study Period: not specified Tool: Duo classifier built on top of the cost-sensitive variant of the random forest algorithm Focus: Prioritizes patient enrollment based on the likelihood of progression in a trial.
Beaulieu et al., 2021 [100]	Recruitment	The PRO-ACT database includes data from 23 clinical trials involving 10,700 ALS patients.	Study Period: 2008 to 2015 Tool: A VC-independent gradient boosting machine survival model Focus: Replace vital capacity (VC)-based selection in ALS clinical trials, offering a more inclusive, risk-based approach for COVID-19 patient selection.
Cesario et al., 2021 [83]	Recruitment	No specification. Data collected from high-volume cancer center – Comprehensive Cancer Center at Fondazione Policlinico Universitario A. Gemelli IRCCS.	Study Period: not specified Tool: Digital Research Assistant, developed as a progressive web app Focus: Analyze patients’ clinical and genetic profiles to suggest eligible trials, supporting a personalized medicine approach.
Wilson et al., 2018 [127]	Recruitment	The eight trials recorded basic information about patients screened for trial participation and randomization outcomes.	Study Period: not specified Tool: The SEAR (Screened, Eligible, Approached, Randomized) rule-based model Focus: Encourages the collection of information to identify recruitment obstacles and facilitate improvements to the recruitment process.
O’Regan et al., 2023 [69]	Recruitment	From ClinicalTrials.gov and supporting the TARGET National study, which aims to profile up to 6000 patients over five years genomically.	Study Period: July 2021 to July 2026 Tool: The Digital ECMT Cancer Trial Matching Tool-NLP Focus: Extract, normalize, and rank trial eligibility criteria, integrating cancer type, genetic mutation, and intervention mechanism data and assigning rule-based scores to match.
Hardy-Abeloos et al., 2023 [128]	Recruitment	476 breast cancer patients, 259 patients (54%) had in-person consultations, while 217 patients (46%) used telemedicine.	Study Period: 1 June 2020 to 13 May 2021 Tool: Univariate and multivariate logistic regression models Focus: Identify factors associated with patient preference for an initial consult via telemedicine and correlation with clinical trial enrollment.
Nievas et al., 2024 [70]	Recruitment	Obtained from the SIGIR dataset, in total, 2000 patient-trial pairs.	Study Period: not specified Tool: Both proprietary (GPT-3.5, GPT-4) and open-source large language models (LLMs) (LLAMA 7B, 13B, 70B) Focus: Systematically examine the efficacy of models in matching patients with suitable clinical trials.
Kusa et al., 2023 [71]	Recruitment	The study used TREC Clinical Trials datasets, sourced from ClinicalTrials.gov, comprising 375,580 clinical trial documents, with the 2021 cohort containing 75 patient cases and 35,832 relevance judgments, while the 2022 cohort included 50 patient cases and 35,394 relevance judgments.	Study Period: not specified, but the data covered from 2021 and 2022 Tool: Named entity recognition and negation detection Focus: Classify patient descriptions and CT eligibility criteria into current, past, and family medical conditions, helping match the patient information to the criteria section.
Shi et al., 2020 [113]	Recruitment	The dataset included 119 enrolled patients across nine clinical studies, with an additional 86,292 individuals from SHARE used for model validation. Patient information, including diagnoses (ICD-10 codes), medical procedures (OPCS-4 codes), prescriptions (BNF codes), and laboratory test results, was sourced from NHS Scotland’s EHRs.	Study Period: not specified, data covered between 2010 and 2017 Tool: NLP Focus: Analyze free-text EHR data, improving patient ranking accuracy and targeting patients eligible for clinical studies.
Dhayne et al., 2021 [129]	Recruitment	The dataset included 10,000 randomly selected patients from MIMIC-III and 10,000 clinical trials sourced from ClinicalTrials.gov.	Study Period: not specified Tool: EMR2vec-a platform that customizes advanced NLP, machine learning, and semantic web techniques Focus: Link potential patients to suitable clinical trials and enhance recruitment efficiency.
White et al., 2024 [130]	Retention	The cohort consisted of 100 participants diagnosed with Major Depressive Disorder (MDD) who were recruited from a prior study, the RADAR-MDD study, at the London site.	Study Period: April/ May 2021 to September 2021 Tool: The RADAR-based system-remote symptom-tracking platform Focus: Integrate a smartphone app and a Fitbit wearable over a 12-week period, providing behavioral theory-based notifications, real-time progress tracking, and direct researcher contact options to improve engagement.
Hulstaert et al., 2024 [131]	Recruitment	No specification of patient numbers, but the target patient population for clinical trials was in two specific indications: inflammatory bowel disease (IBD) and multiple myeloma (MM).	Study Period: 4 data sources: (1) 2016–present; (2) 1990–present; (3) initiated in 2000; (4) initiated in 1996 Tool: EMR2vec-a platform that customizes advanced NLP, machine learning, and semantic web techniques. Focus: Using a Poisson regression-based predictive framework enhanced with Random Forest and XGBoost models to predict site-level patient enrollment by analyzing historical performance, investigator experience, patient availability, and site characteristics to identify high-performing sites efficiently.
Zeng et al., 2020 [45]	Recruitment	The dataset contains 38,341 eligibility criteria texts from clinical trials, 22,962 eligibility criteria texts in the training set, and 7682 and 7697 texts in the validation and test sets, respectively.	Study Period: started in 2019 Tool: An ensemble learning model combining five deep learning-based NLP models (BERT, RoBerta, XLNet, ERNIE, ELECTRA) Focus: To automatically classify clinical trial eligibility criteria.
Lee et al., 2024 [132]	Recruitment	From the Mount Sinai Data Warehouse (MSDW) and VieCure, covering 3475 clinical trials involving conditions such as lung cancer, prostate cancer, breast cancer, multiple myeloma, ulcerative colitis, Crohn’s disease, and sickle cell anemia, the dataset included 640 unique eligibility criteria attributes.	Study Period: not specified Tool: A rule-based cohort selection pipeline Focus: Automate the process of identifying eligible patients within EHRs.
Liu et al., 2021 [97]	Recruitment	Clinical trial eligibility criteria were extracted from 352,110 trials, encompassing 3844 unique conditions and 3106 unique interventions.	Study Period: January 2011 to February 2020 Tool: Trial Pathfinder – an NLP-based computational framework Focus: Evaluate the effects of different eligibility criteria in oncology clinical trials using real-world data and simulation methods.
Jreich et al., 2024 [133]	Recruitment	33 research professionals from 13 hospitals across nine clinical research networks in the UK, including principal investigators, research nurses, data managers, and clinical trial practitioners.	Study Period: Flatiron Health Database: January 1, 2011 to February 2022 & Optum’s EHR Database: January 1, 2011 to June 2020 Tool: TRACAT (Trial Rating and Complexity Assessment Tool) a rule-based decision-support system Focus: Evaluate clinical trial complexity, resource demands, and operational feasibility.
Chen et al., 2021 [86]	Retention	731 current smokers, with 13% identifying as Black and 73% as women, from search engines, social media, smokefree.gov, and ResearchMatch.	Study Period: July 2017 to March 2019 Tool: Messages recommender system Focus: Provide personalized smoking cessation messages using collaborative filtering and content-based ranking.
Gardner et al., 2022 [72]	Recruitment	To assess clinical trial-matching performance, the study used 10 synthetic patient cases representing small cell lung cancer (SCLC) and non-small cell lung cancer (NSCLC).	Study Period: not specified Tool: NAVIFY Clinical Trial Match tool-cloud-based clinical workflow decision support Focus: Match cancer patients to clinical trials.
Alexander et al., 2020 [73]	Recruitment	The dataset was sourced from patients at an Australian specialist cancer hospital, specifically from the Thoracic Malignancies Cohort (TMC) study. A total of 102 lung cancer patients were evaluated for eligibility across 10 Phase I–III clinical trials.	Study Period: no specification, but patients diagnosed between 2012 and 2018 Tool: NLP-based eligibility criteria processing and extracting tool Focus: Match lung cancer patients to clinical trials at an Australian cancer hospital.
Wang et al., 2024 [81]	Recruitment	The dataset comprised 1053 consecutive inpatients referred to the Liver Tumor Center at Nanfang Hospital, Southern Medical University in Guangzhou, China, between January and December 2019.	Study Period: no specification, but the dataset was derived from January 2019 to December 2019 Tool: NLP-based screening tool Focus: Extract and analyze medical data from electronic health records (EHRs) and match patients with hepatocellular carcinoma (HCC) to appropriate clinical trials.
Liu et al., 2022 [134]	Recruitment	57 participants (with 38 having used ATLAS before, 32 of them self-identified as programmers, 18 of them self-identified as practicing physicians or clinical practitioners, and 12 of them as research staff), including 278 eligibility criteria from 20 clinical trials randomly sampled from ClinicalTrials.gov.	Study Period: not specified, but during the 2019 OHDSI Fall Symposium Tool: Criteria2Query (C2Q), an NLP-based text-converting tool Focus: Convert eligibility criteria into structured OMOP Common Data Model (CDM)-based database queries.
Selker et al., 2018 [135]	Recruitment	There was no specification of participant number, but patient data was collected from 6 hospitals’ emergency departments (EDs), who were over the age of 30 and had 12-lead electrocardiograms (ECGs) performed with acute coronary syndromes.	Study Period: start and end not specified, but it’s a three-month assessment Tool: BERT-based Acute Cardiac Ischemia Time-Insensitive Predictive Instrument (ACI-TIPI) Focus: Use electrocardiographic data to improve the accuracy of identifying candidates for clinical trials in acute coronary syndromes.
Mellem et al., 2021 [136]	Retention	215 patients with schizophrenia, with 113 patients in the 15 mg/day paliperidone arm and 102 in the placebo arm.	Study Period: no specification of start and end day, but the trial was held for 6 weeks Tool: Personalized Advantage Index (PAI) modeling and Bayesian Rule Lists (BRL) Focus: Identify schizophrenia patients more responsive to paliperidone, improving treatment effect size and reducing required sample sizes.
Ott et al., 2019 [98]	Recruitment	No cohort specification, but patients were identified for clinical trials using electronic health records (EHRs) by integrating HL7 Clinical Document Architecture (CDA) templates.	Study Period: not specified Tool: HL7 V3-based automated patient-trial matching tool Focus: Define and apply clinical trial eligibility criteria using HL7 V3 templates, enhancing patient selection from EHRs.
Gulden et al., 2019 [99]	Recruitment	The dataset includes 50 clinical trials registered on ClinicalTrials.gov, with a total of 1120 eligibility criteria.	Study Period: not specified Tool: Atlassian Confluence platform-a web-based tool for collaborative editing of documents Focus: Analyzes the prevalence of structured data elements in clinical trial eligibility criteria to improve automated patient recruitment.
Hao et al., 2019 [108]	Recruitment	Synthetic cohort generated via the Synthea Patient Generator: 7889 patients with 846,073 condition values across 136 LOINC codes.	Study Period: not specified Tool: An automated regional search tool Focus: Use collaborative filtering to recommend cost-efficient examinations by computing a “utilized cost” for each medical exam, thus reducing expenses and accelerating participant recruitment in clinical trials.
Kempf et al., 2023 [101]	Recruitment	205,977 patients with cancer from the Greater Paris University Hospital (APHP) clinical data warehouse (the study focuses specifically on patients eligible for 15 randomly selected phase I–IV urology clinical trials conducted between 2016 and 2021.	Study Period: no specification, but data were collected from 2016 to 2021 Tool: PENELOPE- an Observational Medical Outcomes Partnership (OMOP) common data model (CDM) Focus: Translate and execute eligibility queries, increasing their compatibility and effectiveness of patient prescreening.
Vydiswaran et al., 2019 [57]	Recruitment	288 longitudinal patient records annotated for 13 selection criteria derived from real clinical trials; the dataset was split into 202 training records and 86 test records.	Study Period: not specified Tool: A combined pattern-based, knowledge-intensive, and feature-weighting technique Focus: Identify “met” and “not met” cases to automate cohort selection.
Bucalo et al., 2021 [137]	Recruitment	The data includes two clinical trials: COMPLEMENT-1 (2017), which enrolled 14 patients, and BioItaLEE (2018), which enrolled 13 patients.	Study Period: no specification, but the data was collected during 2017 (COMPLEEMENT-1 study) & patients enrolled during 2018 (BioltaLEE study) Tool: NLP-based information extracting tool Focus: Identifying patients through automated inclusion/exclusion criteria queries
Stevens et al., 2021 [138]	Recruitment	190,609 patients from the US Department of Veterans Affairs Health System, aged 50 or older, with no Clostridioides difficile infection (CDI) in the year before the study.	Study Period: January 1, 2009 to December 3,1 2013 Tool: A two-stage predictive algorithm Focus: Identify patients at high risk of developing CDI between months 2 and 12 post-screening for enrollment in Clostridioides difficile vaccine clinical trials.
Kehl et al., 2024 [74]	Recruitment	The data contains 2150 patients with solid tumors who had undergone next-generation sequencing; 65% were female with a median age of 63 years.	Study Period: April 2022 to January 2023 Tool: A genomic-based patient-matching tool with a neural network prediction and MatchMiner platform Focus: Analyze radiology reports to predict patients likely to change treatment within 30 days, flagging potential candidates for recruitment.
Stemerman et al., 2021 [58]	Recruitment	The data contains 1209 EMS electronic health records (EHRs) patient records.	Study Period: December 2017 to July 2018 Tool: NLP-based patient identification Focus: Classify patients for prehospital clinical trials based on EMS clinical notes.
Kanbar et al., 2022 [54]	Recruitment	A history of surgery included in the training set increased from 102 patients on April 10, 2016, to 195 patients on October 6, 2019. & ACTES Cohort: pediatric emergency department patients screened for clinical trial eligibility from a pool of 170,000 annual visits.	Study Period: April 12, 2016 to October 6, 2019 Tool: EHR-based NLP Focus: Provides seamless, real-time integration of AI technology to provide patient enrollment decision support and improve patient care by efficiently identifying surgical candidates.
Marcath et al., 2021 [112]	Recruitment	No specification of participant number, but the data were identified based on high prevalence rates of DDI (Drug-Drug Interactions) among trial participants.	Study Period: not specified Tool: interview-based, a drug-drug-interaction screening tool Focus: Detect DDI during oncology clinical trial.
Calaprice-Whitty et al., 2020 [65]	Recruitment	Three oncology protocols, encompassing 48,124 patients, were selected for this study from those who had completed enrollment at CBCC within the past four years.	Study Period: May 2018 to June 2018 Tool: Mendel.ai – an NLP-based text recognition tool Focus: Prescreen patient records against trial eligibility criteria, increasing the number of correctly identified candidates.
Cai et al., 2021 [51]	Recruitment	A total of 3359 patients from Brigham and Women’s Hospital and 642 from Faulkner Hospital.	Study Period: 2016 to 2020 Tool: An ensemble machine learning algorithm (SMALL) that integrates random forest (RF) and logistic regression with LASSO Focus: Improve the efficiency of patient eligibility screening.
Chen et al., 2020 [139]	Recruitment	No specification of participant number; the data were collected from Stanford Cancer Center patients’ genomic data, obtained from formalin-fixed paraffin-embedded (FFPE) tissue blocks, and underwent targeted next-generation sequencing (NGS).	Study Period: 2014 to 2019 Tool: An automated algorithm Focus: Match patients to precision medicine clinical trials based on genetic biomarkers.
Borno et al., 2021 [111]	Recruitment	52 evaluable patients with newly diagnosed high-risk prostate cancer (Gleason Score ≥8).	Study Period: May 2019 to September 2019 Tool: A questionnaire-driven matching and pathology reporting tool Focus: Improve recruitment of diverse, high-risk prostate cancer patients into clinical trials at the time of diagnosis, by incorporating rule-based logic and natural language processing to personalize clinical trial recommendations.
Getz et al., 2020 [90]	Retention	2976 participants across 23 clinical trials, including 260,000 dosing observations, focusing on psychiatric, neurological, and neuromuscular diseases.	Study Period: December 2016 to May 2019 Tool: AiCure, a platform combining artificial intelligence (integrates advanced OCR, clinical NLP, knowledge-based reasoning, semantic search, and continuous human-in-the-loop learning) and virtual patient monitoring Focus: Assess the extent and predictors of intentional dose non-adherence in clinical trials.
Adams et al., 2022 [87]	Retention	1,183 U.*S. cancer* patients and survivors, predominantly female (72%) and White (70%), with 41% having or surviving breast cancer and 28% in active treatment.	Study Period: July 6 to September 8, 2021 Tool: Remote interventions (like video visits, wearable tech, and virtual informed consent) Focus: Promote patients’ self-reported willingness to enroll in cancer clinical trials.
Theodorou et al., 2024 [103]	Recruitment	Trial site data was gathered from 33,323 sites - 4392 clinical trials.	Study Period: 2016 to 2021 Tool: RAMM (Fair Ranking with Missing Modalities)-a deep reinforcement learning (RL) framework Focus: Enhance both enrollment efficiency and participant diversity in clinical trials.
Yuan et al., 2023 [75]	Recruitment	No specification of the cohort; the model evaluated 125 eligibility criteria from ClinicalTrials.gov across various disease domains and 52 user-entered criteria.	Study Period: not specified Tool: Criteria2Query system- natural language interface (NLI) that translates free-text clinical trial eligibility criteria into structured, executable SQL queries Focus: Match patients to suitable clinical trials while preserving data privacy.
Dobbins et al., 2022 [35]	Recruitment	The Leaf Clinical Trials (LCT) corpus contains 1006 annotated clinical trial eligibility criteria.	Study Period: no specification of end date; the start date is January 1, 2018 Tool: NLP-based text transforming tool Focus: Transform free-text eligibility criteria from electronic health records (EHR) into structured database queries, enabling more effective identification of patients who qualify for clinical trials.
Dobbins et al., 2023 [140]	Recruitment	The system was tested on eight clinical trials spanning cardiology, COVID-19, Crohn’s disease, multiple sclerosis (MS), diabetes mellitus, hepatitis C, and cancer, involving a total of 427 enrolled patients at the University of Washington.	Study Period: January 2017 to December 2021 Tool: An NLP-driven, rule-based query generation system Focus: Automate eligibility screening.
Bustos et al., 2018 [47]	Recruitment	Over 6 million short clinical statements extracted from 49,201 cancer clinical trial protocols on ClinicalTrials.gov	Study Period: 2000 to 2018 Tool: Deep learning model Focus: Predict whether clinical statements represent inclusion or exclusion criteria in cancer clinical trials.
Uspenskaya-Cadoz et al., 2019 [141]	Recruitment	88,298,289 subjects aged 50–85 years, with (1) the positive cohort (667,288 individuals with at least one Alzheimer’s disease (AD) diagnosis or AD treatment record), (2) the negative cohort (3,670,254 individuals without AD, matched based on prevalence rates), and (3) the scoring cohort (72,670,283 individuals with recent medical records but no AD diagnosis).	Study Period: January 2010 to July 2018 Tool: A machine learning classification model, including Logistic Regression, Decision Trees, Random Forest, and Gradient Boosted Trees (GBT) Focus: Identify undiagnosed prodromal Alzheimer’s Disease (AD) patients in the general population to aid early detection and improve clinical trial enrollment.
Xu et al., 2023 [142]	Recruitment	Two Phase III clinical trials were analyzed: (1) Donepezil trial (NCT00478205) for Alzheimer’s disease (AD), and (2) Bevacizumab trial (NCT00112918) for colorectal cancer (CRC) (a) 4,998 patients with Alzheimer’s disease treated with donepezil and (b) 739 colorectal cancer patients treated with FOLFOX4 Covering approximately 16.8 million patients with linked Medicaid, Medicare claims, cancer registries, and vital statistics.	Study Period: not specified, but there were 30 days between application and follow-up Tool: A supervised Poisson factor analysis (SPFA) Focus: Identify subphenotypes (patient subgroups) based on clinical characteristics and risk of severe adverse events (SAEs).
Do et al., 2024 [85]	Recruitment	No specification of participant number, but the data was collected from the VA’s Corporate Data Warehouse (CDW) for comprehensive patient records, the VA Cancer Registry for oncology-specific data, and the VA National Precision Oncology Program (NPOP) for genomic sequencing results.	Study Period: not specified Tool: NLP-based matching tool Focus: Enhance precision in patient-trial matching.
Hassanzadeh et al., 2020 [76]	Recruitment	The dataset from the 2018 n2c2 shared task (Track 1) focused on cohort selection for clinical trials.	Study Period: not specified Tool: NLP-based semantic vector representations and a multi-layer perceptron (MLP) model Focus: Extract key eligibility criteria from unstructured EHR clinical documents.
Jin et al., 2024 [77]	Recruitment	There are three publicly available cohorts: SIGIR 2016 (59 patients), TREC 2021 (75 patients), and TREC 2022 (50 patients).	Study Period: not specified Tool: NLP-based matching tool Focus: Automate patient-trial matching to improve efficiency and accuracy, achieving near-human performance in predicting eligibility and reducing clinical trial screening time.
Chen et al., 2019 [114]	Recruitment	Patient records from the 2018 National NLP Clinical Challenge (n2c2) consist of 288 longitudinal patient records annotated by medical experts for 13 selection criteria related to personal and clinical history.	Study Period: not specified Tool: Medical Knowledge-infused Convolutional Neural Network (MKCNN) Focus: Improve cohort selection by integrating clinical text representation with medical knowledge and determining the eligibility status of patients based on their electronic health records.
Chorev et al., 2023 [143]	Recruitment	The cohort was composed of treatment-naïve adults aged 50–100 years, 1720 eyes from 1612 patients diagnosed with neovascular age-related macular degeneration (active MNV) across 10 clinics in the UK.	Study Period: 18 December 2019 to 4 August 2021 Tool: A multi-modal AI-driven cohort selection tool with deep learning (ResNet50) and logistic regression Focus: Identify patients likely to have persistent macular fluid after treatment and enroll these patients.
Sun et al., 2023 [36]	Recruitment	The dataset comprises 1508 AD clinical trials sourced from ClinicalTrials.gov, with 300 trials manually annotated for evaluation.	Study Period: not specified Tool: CLAMP (Clinical Language Annotation, Modeling, and Processing) and SapBERT (a deep learning-based biomedical entity normalization model) Focus: Enhances patient-trial matching efficiency by achieving high accuracy in entity recognition and normalization.
Tissot et al., 2020 [59]	Recruitment	Electronic health record (EHR) data from critical care units across five major Biomedical Research Centers (BRCs): Cambridge, Guys/Kings/St Thomas,’ Imperial, Oxford, and UCLH. Standardized structured data, including 108 descriptors related to hospitals, units, patients, and admissions, as well as 154 time-varying variables such as vital signs, lab results, nursing activities, and medication administration.	Study Period: June 2014 to December 2015 Tool: NLP-driven CogStack platform Focus: Reduce manual screening efforts and improve trial enrollment efficiency by combining structured EHR data with unstructured clinical text and applying multiple Natural Language Processing (NLP) components to extract and contextualize clinical concepts.
Delorme et al., 2021 [60]	Recruitment	Electronic health records (EHRs) from patients enrolled in Phase I or II oncology trials for various tumor types at DITEP and lung cancer at the Medical Oncology Department of Gustave Roussy, France. Out of 111,091 total documents, 56,924 were long reports (TC1), including 1858 records specifically related to consultations for early-phase clinical trial inclusion (TC2).	Study Period: September 2012 to July 2020 Tool: NLP and random forest Focus: Screen the patient consultation report and improve patient selection in Phase I and II oncology trials, reducing screen failures and enhancing trial efficiency.
Xu et al., 2023 [78]	Recruitment	460,952 clinical trials with 122,706 cancer-related trials and 2227 manually annotated trials.	Study Period: not specified Tool: NLP-based eligibility matching tool Focus: Enhances patient-trial matching accuracy by integrating structured genomic profiles, reducing manual workload and screening time while improving trial enrollment efficiency.
Gligorijevic et al., 2019 [105]	Recruitment	The dataset includes 2618 clinical studies spanning over 20 years, incorporating investigator data, EHR data, and public study data.	Study Period: no specification, but the collected data spans the past 20 years. Tool: DeepMatch (DM)-an EHR-based matching tool Focus: Enhance clinical trial site selection, reducing recruitment delays and improving investigator ranking accuracy.
Roy et al., 2021 [91]	Retention	This data was derived from two Phase II trials: schizophrenia (NCT03351244) and Attenuated Psychosis Syndrome (NCT03230097); in total, it consisted of 235 participants.	Study Period: not specified Tool: AiCure – a computer vision-based mobile application Focus: Visually confirm the dosing events and transfer and document the adherence data in real-time.
Kim et al., 2022 [37]	Recruitment	The data was searched under “Prostatic Neoplasms” on August 17th, 2021, on ClinicalTrials.gov., including 483 clinical trials.	Study Period: no specification of start date, end date (data collection) August 17th, 2021 Tool: Google Healthcare Natural Language API-NLP-based system Focus: Extract, classify, and structure free-text eligibility criteria into a machine-readable format.
Tian et al., 2021 [144]	Recruitment	Free-text eligibility criteria of 13 phase III and IV AD clinical trials for existing Food and Drug Administration (FDA) approved AD drugs.	Study Period: not specified Tool: A Multi-Input Multi-Output (MIMO) sequence labeling model Focus: Extract structured eligibility criteria from unstructured clinical trial text and translate these criteria into computable queries for Electronic Health Records (EHR).
Fang et al., 2021 [39]	Recruitment	No specification of participant number; the data were derived from the ARCADIA trial (NCT03192215) on patients with atrial cardiopathy and cryptogenic stroke.	Study Period: not specified; the study is still ongoing Tool: An NLP and Criteria2Query-based human-computer collaborative approach Focus: Extract and map eligibility concepts, followed by manual refinement to remove non-queryable or redundant criteria, simplifying eligibility criteria.
Wang et al., 2024 [145]	Recruitment	The dataset consists of 2,000 patient records, 1926 of which contain clinical notes collected from the Epic Clarity EHR database.	Study Period: not specified Tool: NLP and XGBoost-based patient phenotyping tool Focus: Automate patient phenotyping to identify AD (Atopic Dermatitis) patients from EHRs.
Alexander et al., 2022 [146]	Recruitment	No specification of participant number; data were derived from three sources: the VHA Corporate Data Warehouse (CDW), the Veterans Health Information Systems and Technology Architecture (VistA), and the eligibility telephone screen surveys of potential GenoVA Study participants at baseline.	Study Period: from June 2020 to November 2021 Tool: A computable EHR classifier Focus: Pre-screen the exclusion criteria for the GenoVA clinical trial.
Matos et al., 2021 [147]	Recruitment	799 patients with advanced solid tumors who were enrolled in phase I clinical trials at Vall d’Hebron Institute of Oncology (VHIO) between January 2011 and March 2020, 518 patients receiving immune checkpoint inhibitors (ICIs), and 281 receiving targeted agents (TAs).	Study Period: January 2011 to March 2020 Tool: Phase I Prognostic Online (PIPO) - a web-based prognostic calculator Focus: Predicts the overall Survival rate and 3-month survival probabilities for patients being considered for early-phase oncology trials.
Meystre et al., 2023 [61]	Recruitment	400 patients, including at least 100, were enrolled in one of the five selected cardiovascular and oncology clinical trials.	Study Period: 2018 to 2019 Tool: Generalized additive models (GAMs) Focus: Calculate individual risk scores and recruit high-risk participants.
Seixas et al., 2023 [109]	Retention	A total sample of 100 participants (50 each in the control and intervention arms) was recruited over a period of 18 months during the height of the COVID-19 pandemic.	Study Period: not specified Tool: The PREDHiCT app-an e-persuasive mobile application Focus: Provide personalized education, clinical trial navigation, health tracking, and AI-curated newsfeeds to improve clinical trial literacy and engagement.
Mezlini et al., 2023 [55]	Recruitment	The data was recruited from the Evidation Health platform; 840 high-risk people were tracked, with 104 confirmed COVID-19 cases.	Study Period: 2020.6.15 to 2020.8.3 and 2020.11.5 to 2021.4.15 Tool: Generalized additive models (GAMs) Focus: Calculate individual risk scores and recruit high-risk participants.
Langford et al., 2020 [148]	Recruitment	The A4 screening dataset includes 4,486 participants, of whom 1323 (29%) were identified as Aβ-positive (Aβ+).	Study Period: 2018 to 2020 Tool: XGBoost predictive model Focus: Predict amyloid positivity (Aβ+) based on demographic, genetic, and cognitive variables to reduce the number of PET scans needed for recruitment, thus cutting costs and improving efficiency.
Ansoborlo et al., 2023 [62]	Recruitment	The data utilized 640 anonymized MTM (multidisciplinary team meetings) reports from a French university hospital.	Study Period: 2018 to 2020 Tool: NLP-based pre-screening tool Focus: Automate clinical trial prescreening to reduce manual workload, minimize delays in recruitment, and enhance the precision of trial enrollment processes.
Ma et al., 2023 [110]	Recruitment	The cohort consisted of 2877 unique patients identified by the EHR algorithm over the study period. These patients were on average 78 years old, predominantly female (58.3%), White (64.9%), and primarily insured by Medicare (91.4%).	Study Period: 2019.1.1 to 2021.1.31 Tool: An EHR-based notification system Focus: Prompt clinical care providers to engage in advance care planning (ACP) conversations and based on the conversation content, refer patients to a clinical trial.
Ni et al., 2019 [56]	Recruitment	46,612 patients visit the pediatric emergency department (ED) at Cincinnati Children’s Hospital Medical Center (CCHMC), a Level 1 trauma center with over 70,000 patient visits annually.	Study Period: October 1, 2017 to September 30, 2018 Tool: Automated Clinical Trial Eligibility Screener (ACTES) that integrates Natural Language Processing (NLP) and machine learning (no specification of details) Focus: Reduce manual workload and improve recruitment efficiency.
Delozier et al., 2021 [95]	Recruitment	149 patients identified at Vanderbilt University Medical Center were experiencing rare adverse drug events (ADEs).	Study Period: 2017.10.1 to 2018.9.30 Tool: NLP-based real-time clinical note monitoring system Focus: Detect Stevens-Johnson syndrome, trigger alerts, and notify study personnel for rapid case trial assessment and enrollment.
Craig et al., 2023 [92]	Recruitment	The study covered retrospective medical (e.g., commercial fully insured, Medicare, and Medicaid) claims (*N* = 6,348,500), pharmacy claims (*N* = 127,407,048), personal (e.g., first name, surname), and demographic data (e.g., R/E, zip code) claims.	Study Period: not specified Tool: Integrates machine learning (ML), Bayesian inference, and statistical models Focus: Ensure that clinical trial findings are more generalizable and equitable across racial and ethnic groups.
Ansart et al., 2020 [52]	Recruitment	The data contained three cohorts: ADNI-MCI (596 MCI patients with 62.9% amyloid positive), ADNI-CN (431 cognitively normal patients with 37.6% positivity), and INSIGHT-preAD (318 asymptomatic patients with subjective memory issues and 27.7% positivity).	Study Period: no specification of end date; the start time is 2019.1 Tool: Random forest-based pre-screening algorithm Focus: Predict amyloid positive using cognitive, genetic, and socio-demographic data, decreasing recruiting PET scan costs.
Lauffenburger et al., 2021 [88]	Retention	The data contained 60 patient records with type 2 diabetes who have suboptimal glycemic control (HbA1c ≥7.5%) and are prescribed 1–3 daily oral diabetes medications derived from Brigham and Women’s Hospital (BWH), an academic medical center in Boston, Massachusetts, USA.	Study Period: 2021.2.4 to 2021.8.4 Tool: A reinforcement learning-based text messaging intervention Focus: Personalizes daily medication reminders based on real-time adherence data from electronic pill bottles.
Abiodun et al., 2022 [93]	Retention	No specific cohort details; part of the cohort is COVID-19 patients.	Study Period: not specified Tool: A bagged SVM and ANN classifiers Focus: Analyze physiological data collected from wearable sensors and visualize the data to categorize participants as fit, supporting real-time remote monitoring of patient status in clinical trials and enhancing treatment adherence.
Ismail et al., 2023 [102]	Recruitment	No specification of participant number; the data covered oncology trials (breast and lung cancer) and cardiovascular studies (heart failure, hypertension).	Study Period: not specified Tool: NLP and RAG-based screening tool Focus: Improve the clinical trial process by reducing bias in patient composition and lowering costs and labor.
Karystianis et al., 2019 [43]	Recruitment	288 longitudinal patient records from the 2018 National NLP Clinical Challenges (n2c2) shared task, including information on diagnoses, treatments, medications, and social history, were annotated to determine if patients met 13 specific eligibility criteria.	Study Period: not specified Tool: A rule-based NLP system Focus: Apply syntactical pattern recognition and manually craft dictionaries to extract trial eligibility criteria from unstructured clinical records.
Idnay et al., 2024 [106]	Recruitment	The study recruited 60 clinical research staff experienced in ADRD clinical research from January to March 2022 through institutional listservs, word of mouth, and professional networks. Eligible participants were 18 years or older, fluent in English, had recruited research participants in the past 12 months, and had at least three months of ADRD clinical research experience.	Study Period: January 2022–March 2022 Tool: NLP-based screening tool-Criteria2Query, Informatics for Integrating Biology and the Bedside [i2b2], and Leaf Focus: Enhance Alzheimer’s disease and related dementias) clinical trial recruitment by automating eligibility screening and improving efficiency.
Lanera et al., 2022 [149]	Recruitment	257 patients diagnosed with knee osteoarthritis. Participants were divided between two trials: Trial A: 120 patients and Trial B: 137 patients.	Study Period: not specified Tool: SuperLearner ML model Focus: Automate the run-in selection for clinical trials, improving patient screening accuracy while reducing costs and delays.
Yang et al., 2023 [46]	Recruitment	The data were derived from PROTECTOR1, a manually annotated database comprising 764 Phase III cancer clinical trials sourced from ClinicalTrials.gov.	Study Period: 2000 to 2017 Tool: A text classification model Focus: Automate the common exclusion criteria classification and reduce manual workload.
Simon et al., 2019 [150]	Recruitment	The data covered pediatric abdominal pain in 11 Northern California emergency departments, patients 5–20 years of age, and 14,626 ED visits at 11 Kaiser Permanente Northern California (KPNC) hospitals.	Study Period: 2016.10 to 2018.1 Tool: Prospective Intelligence System for Clinical Emergency Services (PISCES)-a real-time text message alert tool Focus: Send automated text message alerts to emergency physicians for potential study enrollment.
Chang et al., 2023 [79]	Recruitment	Data was collected from six major stroke clinical trials: NCT03735979, NCT03805308, NCT03263117, NCT03496883, NCT03876457, and NCT03545607, which include 825 individuals in total.	Study Period: Tool: FairPM-a deep learning framework (integrated BERT-based embeddings, memory networks, and fairness constraints) Focus: Reduce recruitment bias and create more inclusive clinical trials while maintaining high prediction accuracy
Lobe et al., 2018 [151]	Recruitment	Data was sourced from five oncology clinical trials (breast and esophageal cancer) that faced low recruitment rates: 101 patients from Leipzig University Cancer Center (UCCL), with 212 medical records (doctors’ letters and follow-up documentation).	Study Period: no specification of end date; the study was actively conducted in 2015 Tool: Apache UIMA and cTAKES-NLP-based pipeline Focus: Extract structured information from unstructured medical free text, automating the eligibility screening for oncology trials.
Tian et al., 2021 [40]	Recruitment	The study covered two datasets: the Chia Dataset, which contains 12,409 eligibility criteria from 1,000 Phase IV clinical trials and is annotated with 41,487 distinctive entities across 15 entity types, and the FRD Dataset, which contains approximately 50,000 eligibility criteria from 3,314 randomly selected clinical trials in the United States and is annotated with 15 entity types.	Study Period: late 2010s to early 2020s Tool: An advanced transformer-based NER model Focus: Parse and structure clinical trial eligibility criteria, recognizing their entity types (e.g., condition, drug, procedure).
Xiong et al., 2022 [152]	Recruitment	The study covered two datasets: The N2C2 Dataset contains the medical records of 576 patients. Each patient has 2–5 longitudinal electronic medical records (EMRs) annotated with 13 cohort selection criteria. MIMIC-III Dataset: Contains medical records of 844 patients. The dataset includes phenotype annotations for various conditions, such as advanced cancer, heart disease, and chronic pain.	Study Period: no specification of end date; the start time is 2018 Tool: MRC framework with a cross-criterion attention mechanism Focus: Improve the accuracy of patient selection for clinical trials.
Shafner et al., 2018 [96]	Retention	Aggregated data were collected across seven schizophrenia studies; three trials were completed, and four are ongoing, with a total enrollment of 1312 subjects.	Study Period: no specification of start and end; the study duration is 52 weeks Tool: A smartphone platform Focus: Track medication ingestion using visual confirmation technology, providing automated reminders, alarms, dosing schedules, and real-time intervention dashboards.
Vazquez et al., 2020 [153]	Recruitment	The data consisted of 102,510 unique users from ResearchMatch, primarily white females, with a mean age of 35.67 years, and the most common medical conditions being depression, hypertension, and anxiety.	Study Period: not specified, but the dataset includes de-identified information about participants from ResearchMatch Tool: Multiple supervised machine learning classifiers (Random Forest, Logistic Regression, and deep learning Convolutional Neural Network (CNN)) Focus: Predict individuals’ likelihood of participating in clinical trials based on demographic, medical, and behavioral data.
Beattie et al., 2024 [80]	Recruitment	202 longitudinal patient records from the National NLP Clinical Challenges (n2c2) 2018 dataset, annotated by medical professionals and evaluated against 13 eligibility criteria, covering conditions such as diabetes, cardiovascular disease, and substance abuse.	Study Period: not specified Tool: Large Language Models (LLMs)-GPT-3.5 Turbo and GPT-4, with retrieval-augmented generation (RAG) and structured prompt engineering Focus: Automate patient screening for clinical trial matching, achieving high accuracy, reducing manual workload and processing time.
Schwager et al., 2021 [154]	Recruitment	The study included 51,555 acute respiratory distress syndrome (ARDS) patients extracted from the Philips eICU Research Institute (eRI) database, which covered 3 million ICU stays from over 700 U.S. hospitals between 2002 and 2016.	Study Period: 2002 to 2016 Tool: Multiclass gradient boosting models Focus: Predict long-stay ARDS patients to improve patient stratification and optimize recruitment efficiency.
Castillo et al., 2023 [89]	Retention	The dataset included 52 patients with parasitologically confirmed cutaneous leishmaniasis from rural communities in Tumaco, Colombia, with a median age between 20 and 30 years and a predominantly male Afrocolombian composition.	Study Period: not specified Tool: Guaral+ST app-a mobile health tool Focus: Remotely monitor treatment adherence, adverse events, and lesion progression via photographs.
Zimmerman et al., 2018 [155]	Recruitment	The study recruited 422 participants from rural Colombia (five CAPriCORN sites, including Northwestern University, University of Chicago, Rush University Medical Center, NorthShore University HealthSystem, and the Cook County Health and Hospitals System (CCHHS)) and randomized them into intervention (*n* = 212) and control (*n* = 210).	Study Period: not specified Tool: mHealth intervention-a mobile-based app Focus: Implement for over 12 months to facilitate remote symptom tracking and clinician communication, allowing health workers to receive real-time expert feedback and enroll patients more efficiently.

**Figure 2.**
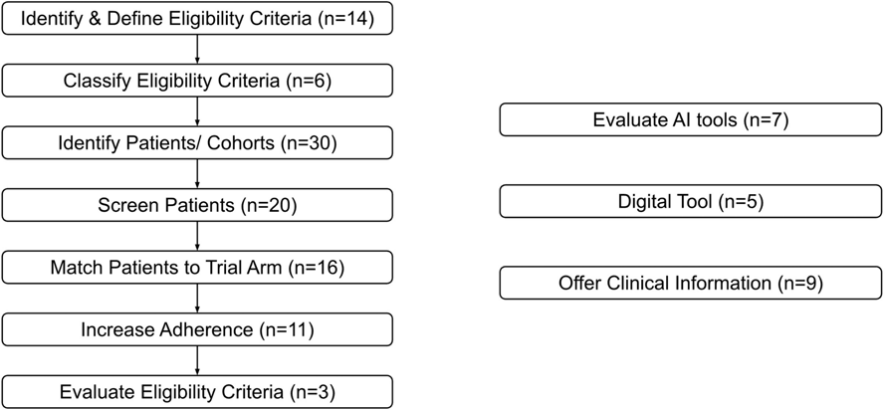
Clinical trial recruitment and retention workflow. The included studies were grouped into ten categories based on the workflow stage targeted by the AI tool.

Figure 2 illustrates a comprehensive, multi-step clinical trial recruitment and retention workflow based on the reviewed studies. The workflow begins with identifying and defining eligibility criteria (*n* = 14), where AI tools extract, structure, and interpret requirements from various sources. Six studies focused on classifying these criteria by converting free text into structured formats. Thirty studies applied AI to identify potential patient cohorts, and 20 used AI for direct patient screening to find individuals likely to meet trial criteria. Patient-trial matching was addressed in 16 studies, using AI to align patient profiles with suitable trials. For retention, 11 studies employed AI to support adherence by addressing barriers. Three studies evaluated past eligibility rules, while eight focused on subprocesses across the trial workflow. Additionally, five studies developed AI-based digital tools to streamline trial processes, and nine used AI to deliver relevant clinical or medical information to stakeholders. Figure 2 summarizes how studies addressed the full recruitment and retention process in clinical trials. Details are in Supplementary Material 4 (*Appendix Table 3.summary of AI tasks, data, evaluation metrics*).

#### AI for eligibility assessment

The AI tools for identifying eligibility criteria are designed to extract, structure, and interpret eligibility requirements. These tools commonly take as input the free-text eligibility criteria from ClinicalTrials.gov [31–40] and unstructured or structured data from electronic health records (EHRs) [41–43]. The AI approaches in this category frequently rely on NLP techniques such as rule-based logic [31,33,41–43], lexicon-enhanced pipelines [31], named entity recognition (NER) [31,32,34–38,40], the concept normalization to standardized vocabularies like OMOP CDM or UMLS [33,34,36,39], and other tools like Apache UIMA, CLAMP, Criteria2Query [31,33,34,36,39,42] for better structured annotation and entity mapping. Nevertheless, the transformer-based models, including BERT, SciBERT, PubMedBERT, and SapBERT, are also used for entity recognition and relation extraction tasks [35,36,38,40]. These approaches typically yield structured outputs such as computable cohort definitions [33,39], entity-attribute relations [35], condition-drug-procedure mappings [36–38], annotated named entities or eligibility criteria tokens [31,35,36,40], and alerts from user interfaces [32,42].

The AI tools that classify eligibility criteria are designed to categorize free-text eligibility statements into structured, interpretable formats. These models typically leverage transformer-based NLP architectures, such as BERT-family variants with ensemble frameworks to improve classification performance [44–46]. Some tools integrate more advanced machine learning algorithms like LightGBM [45] or custom preprocessing pipelines with word embeddings (Word2Vec and FastText) for better phrase detection and classification [47]. This yielded a wide variety of outputs. Some generate predicted category labels spanning dozens of predefined eligibility-related classes [44,47] or binary exclusion indicators for predefined conditions, like prior malignancy, autoimmune disease, or infections [46]. Some other tools convert eligibility text into structured clinical concepts mapped to standardized vocabularies, such as OMOP CDM [48,49], and offer user interfaces for further manual classification, including editing, reviewing, or querying eligibility logic.

#### AI for patient identification and trial matching

AI tools used for direct patient screening aim to efficiently identify individuals or cohorts who are likely to meet key clinical trial eligibility criteria. These tools apply a wide spectrum of methodologies, ranging from classical statistical models like logistic regression [50–52], ensemble learning methods such as random forests, LASSO, and gradient-boosted trees [51,53–56], to more advanced NLP pipelines incorporating NER, rule-based logic, TF-IDF, and transformer-based architectures like GPT-4 or hybrid models with retrieval-augmented generation (RAG) [57–62]. The AI approaches in this category include diverse input sources, including structured and unstructured EHR data such as progress notes, lab values, and ICD codes [50–52,54,56–61,63–65], the patient self-reported or survey data [47,50,55], and the medical image or scanning [53,66]. The outputs from these tools are typically binary classification of eligible or ineligible [50–52,54,56–61,63–65] and predicted eligibility scores [50–53,55,61].

AI tools for patient-trial matching are designed to systematically pair patient profiles with appropriate clinical trials. These tools typically input structured and unstructured EHR data [67–81] with explicitly defined eligibility criteria [18,58,98,136] or free-text eligibility criteria description directly derived from ClinicalTrials.gov or curated trial protocols [68,70–73,75–77,79–81]. Methodologies in this category span several AI and machine learning strategies, including transformer-based models [70,75,77,79,80], the NLP-enhanced rule-based pipelines [68,71–73,76,78,81], the semantic similarity approaches [71,75,76], and other machine learning classifiers, including gradient boosting machines and logistic regression [69,74,79]. These AI tools typically generate binary eligibility decisions indicating whether a patient is eligible for each potential trial [68,70,74,77,79,80]; some are more quantitatively advanced with matching scores [70,74,76,79]. Some tools yield ranked trial lists for each patient as output, with confidence levels or visual explanations of inclusion/exclusion features [68,71–73,78,81].

### AI for trial retention and workflow support

AI-driven digital tools can enhance the clinical trial recruitment and retention process at any stage of the workflow. These tools typically operate as front-end systems, such as chatbots [82], digital research assistants [83], and telemedicine [84]/patient tracking platforms [85]. These tools’ inputs vary based on different tasks, including unstructured free-text eligibility criteria extracted from clinical trial databases [82] and structured and unstructured EHR data [83–85]. The AI approaches are typically NLP and deep learning techniques, such as word2vec embeddings and convolutional neural networks (CNN) for chatbot interfaces [82], and the hybrid NLP pipeline for interaction support [83,85].

The AI tools that are designed for increasing patient adherence during the retention stage usually employ a wide range of methodologies to overcome different adherence barriers, including tailored interventions [86–89], predictive scoring for worsening conditions [90–93], and engagement real-time monitoring [93–96]. This type of AI tools includes diverse input sources, depending on different tasks, including self-reported data and survey responses from patients [86,87,90,92,93], structured and unstructured EHR data [88,92–95], the data tracked by mobile app [91,96], and so on. The key methodologies also span widely depending on the tasks, such as rule-based alert systems [94], computer vision-powered platforms and mobile AI apps for real-time dose tracking and fraud detection [91,96], and more advanced predictive modeling [86,90,93] and an NLP system that flags suspected adverse reactions [95].

AI tools for evaluating eligibility criteria are designed to assess, validate, and optimize how trial eligibility rules are constructed or applied in past clinical trials. The approaches include rule-based logic systems [97], NLP pipelines [98], and predictive modeling frameworks [99]. The evaluate AI models category validates or enhances the performance, generalizability, and fairness of AI tools used when enhancing the effectiveness of clinical trial recruitment and retention. The clinical trial databases [100–102], structured EHR data [100,101,103–106], unstructured notes [102,106], and medical images/scans [104] would generally be used depending on the tool’s focus. Their outputs are either predictive accuracy metrics [100–102,104–106] or fairness metrics like demographic parity and equal opportunity [103].

Lastly, AI tools that offer clinical information support and enhance recruitment and retention decision-making by delivering actionable insights to clinicians, researchers, or patients. These AI tools’ input depends on the type of information they offer, often including EHR-derived medical profiles [107–110], free-text clinical reports [111], structured eligibility criteria [107], or self-reported medication data [112]. More details can be found in Supplementary Material 4 (*Appendix Table 3.summary of AI tasks, data, evaluation metrics*).

**Figure 3.**
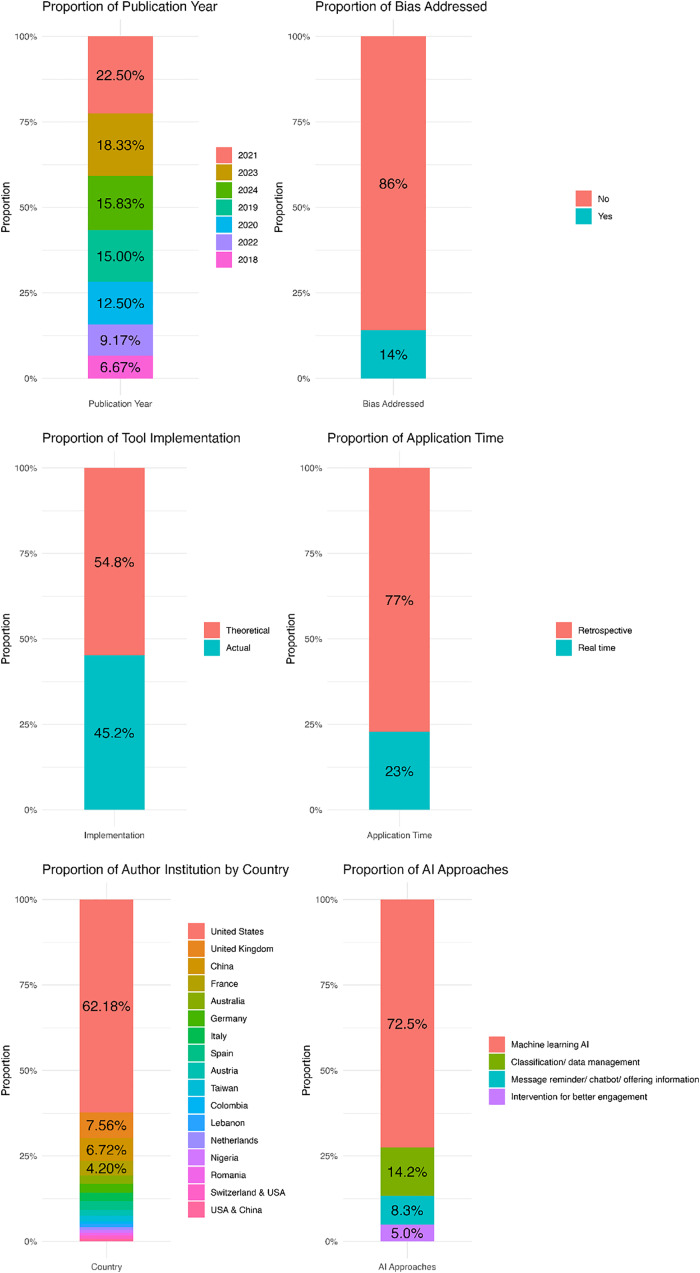
Clinical subject area & publication information. Most of the studies included came from institutions in the USA, and many did not explicitly address biases or disparities. Retrospective and theoretical AI applications predominated among these studies. Machine learning was the most commonly used AI approach.

Figure 3 summarizes key study characteristics, including country, year, disease focus, AI approach, and bias consideration. Most studies were retrospective (77%) and published after 2019 (93.33%), with peaks in 2021 (22.5%), 2023 (18.33%), and 2024 (15.83%). The USA led in publications (62.18%), followed by the UK (7.56%) and China (6.72%). Only 14% addressed bias, and fewer than half (45.2%) applied to actual clinical trials. Most used general machine learning (72.5%), while others relied on rule-based methods. Full details are in Supplementary Material 1 (*Appendix D: Others of the Appendix.additional content*).

### Model performance

#### Primary endpoints

Different clinical trial recruitment and retention stages employed distinct endpoints for evaluating AI models. For patient screening, the true/false positives and negatives are usually defined based on whether the AI tool’s patient eligibility predictions match those determined by manual review or a clinical standard. For identifying eligibility criteria, the true/false positives often refer to whether correctly identified entities match reference annotations. For classifying eligibility criteria, the true positives and negatives usually reflect the correct classification of patient trial arm predictions, while false positives and negatives represent incorrect predictions compared against a manually annotated or gold-standard reference. For patient identification or cohort identification, the true/false positives and negatives are often based on whether the model’s predictions correctly or incorrectly match the manual annotations of potential eligible patients or cohorts.

These results were often reported in papers through model performance metrics, including sensitivity, specificity, accuracy, precision, F-1 score, and AUC. Among the studies we reviewed, for the screen patient category, sensitivity was the most frequently reported metric, appearing in 40% of included papers; however, other performance metrics were less common, with fewer than 30% of papers reporting F1 or AUC. For identifying and defining eligibility criteria, the endpoints usually consisted of the number of records or documents processed during criteria extraction. Sensitivity, specificity, accuracy, precision, F-1 score, and AUC were also commonly used to evaluate this type of AI tool; among these, precision was the most frequently reported metric at 57.1%. Studies focusing on identifying eligible patients or cohorts report the raw number of patients identified alongside model performance measures as a primary endpoint. For this category, sensitivity (30%) was the most frequently reported metric. Similarly, for classifying eligibility criteria, precision (66.7%) and the F-1 score (66.7%) were the most commonly reported endpoints for model evaluation. Details in Supplementary Material 1 (*Appendix D: Others of the Appendix.additional content*). Given that studies targeting different recruitment and retention stages prioritized various performance metrics, we have chosen to present the following metrics in the main text: the sensitivity of screening patients, the precision of identifying eligibility criteria, the sensitivity of identifying patients, and the precision and F-1 score of classifying eligibility criteria.

#### Patient screening

**Figure 4.**
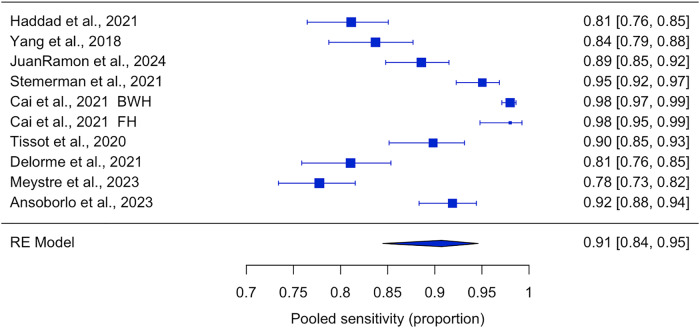
Sensitivity meta-analysis for papers using machine learning for patient screening, with estimates represented by pooled proportions and 95% confidence intervals.

For patient screening, AI tools analyze health data and automatically enroll patients in suitable clinical trials. The efficacy of these tools was assessed through aggregated estimates of key metrics, including specificity, sensitivity, precision, accuracy, F1 score, and AUC. The sensitivity was relatively high, with a pooled estimate of 0.91 [0.85, 0.95] (Figure 4). The pooled specificity was 0.74 [0.33, 0.94]. Precision varied significantly across studies, ranging from 0.22 to 1.00. The pooled F1 score was 0.90 [0.85, 0.94], and the overall classification performance, represented by the area under the ROC curve (AUC), was 0.79 [0.72, 0.85]. The overall pooled accuracy consistently remained high at 0.95 [0.86, 0.98]. Details of all forest plots for the screening patients’ meta-analysis can be found in Supplementary Material 1 (*Appendix C: Additional Figures of the Appendix.additional content*).

#### Identify eligibility criteria

**Figure 5.**
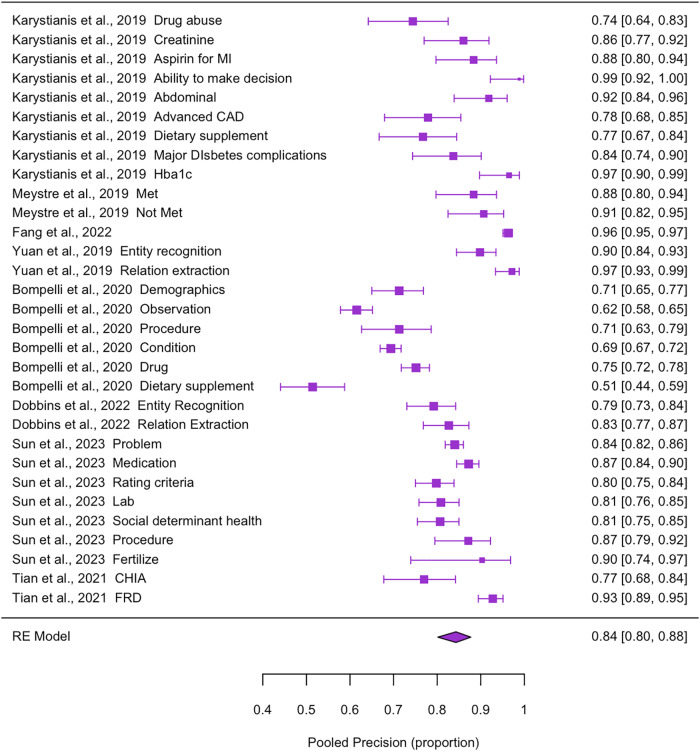
Precision meta-analysis for papers using machine learning to identify eligibility criteria, with estimates represented by pooled proportions and 95% confidence intervals.

To identify and define eligibility criteria, AI tools analyze patients’ clinical records and automatically pinpoint the key medical attributes that align with the trial’s inclusion and exclusion criteria. The model’s performance was also assessed by aggregating key metrics from the included studies. The overall sensitivity was 0.80 [0.76, 0.84]. Pooled precision was relatively higher, with an estimated 0.84 [0.80, 0.88] (Figure 5). Overall accuracy reached 0.93 [0.89, 0.95]. The pooled F1-score was 0.80 [0.77, 0.83]. These results indicate that AI-based tools can systematically and reliably extract eligibility criteria. Detailed forest plots for the meta-analysis of identifying eligibility criteria can be found in Supplementary Material 1 (*Appendix C: Additional Figures of the Appendix.additional content*).

#### Classify eligibility criteria

**Figure 6.**
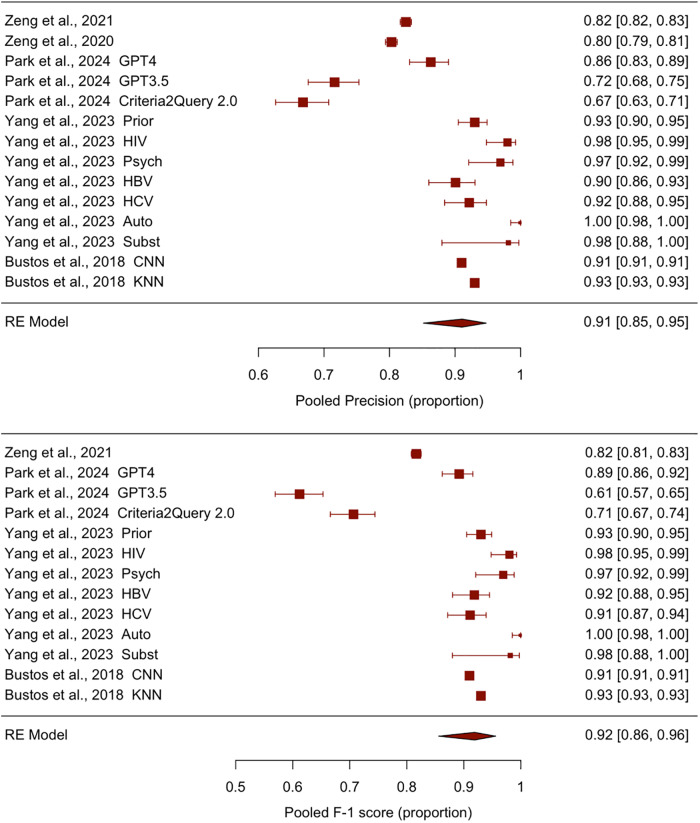
Meta-analysis results for studies using machine learning methods to classify clinical trial eligibility criteria. The upper plot indicates the performance measured using precision, with estimates represented by pooled proportions and 95% confidence intervals. The lower plot indicates the performance measured using F-1 score, with estimates represented by pooled proportions and 95% confidence intervals.

For classifying eligibility criteria, AI tools categorized clinical statements by distinguishing inclusion and exclusion criteria from standard clinical statements and organizing them into various domains. Among the included studies, the performance was encouraging. The pooled sensitivity reached 0.92 [0.84, 0.96], and the aggregated precision was similarly high at 0.91 [0.85, 0.95] (Figure 6). The overall accuracy was 0.94 [0.89, 0.97], and the F1-score was 0.92 [0.86, 0.96] (Figure 6). Ultimately, these findings suggested that the criteria were rarely misclassified, and the AI methods for automating classification proved valuable tools for enhancing clinical trial workflows due to their high reliability and effectiveness. Details of all forest plots for classifying eligibility criteria in the meta-analysis can be found in Supplementary Material 1 (*Appendix C: Additional Figures of the Appendix.additional content*).

#### Patient identification (identify patients/cohorts)

**Figure 7.**
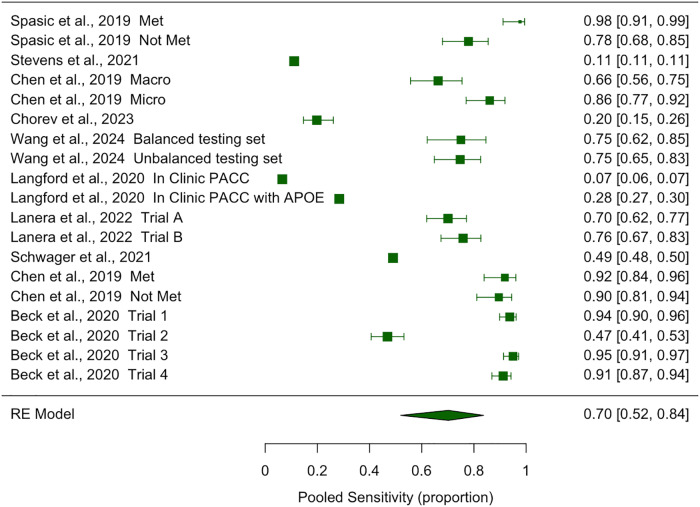
Sensitivity meta-analysis for papers using machine learning to identify patients/cohort, with estimates represented by pooled proportions and 95% confidence intervals.

In studies utilizing machine learning techniques to identify eligible patient cohorts, the pooled sensitivity was 0.70 [0.52, 0.84] (Figure 7). The specificity was 0.79 [0.59, 0.95], and the precision was 0.69 [0.55, 0.80]. The pooled estimation and confidence intervals of sensitivity, specificity, and precision were lower compared to AI models managing other recruitment tasks, with the lower boundary of the interval nearing 0.5, indicating the relatively poor performance of AI-powered techniques in identifying patients and patient cohorts. Overall accuracy reached 0.81 [0.75, 0.86], and the aggregated AUC was 0.74 [0.61, 0.84], with an F1-score of 0.69 [0.32, 0.91]. Collectively, these findings reflect moderate discriminative performance. Details of all forest plots for identifying patients in the meta-analysis can be found in Supplementary Material 1 (*Appendix C: Additional Figures of the Appendix.additional content*). The detailed data for the subsequent recruitment and retention stages that have not undergone meta-analysis can be found in the Supplementary Material 5 (*Appendix Table 4.categorization*).

#### Matching patients

Among the included studies, the accuracy ranges from 0.75 to 0.98, about one third of the studies [68,71,72,76,78] (5/16) output ranked list of clinical trials; and among these five studies, two of them [71,76] evaluated the output relevance with information retrieval (IR) metrics. The included studies proved to solve patient matching barriers. For example, in the study by Jin *et al*. [77], the TrialGPT (a patient labeling model built on GPT-4 and GPT-3.5), along with NLP and rule-based methods, resulted in significant time savings (42.6% overall, with the highest close to 95%). Another FairPM studies [79] addressed fairness concerns by including sensitive attributes such as race and gender in their models. For both patient-criterion matching performance and patient-trial matching performance, FairPM demonstrated drastically reduced demographic parity and equal opportunity values while maintaining slightly reduced accuracy and F1-score compared to the baseline-biased model.

#### Evaluating AI tools

Seven studies developed AI tools that support various stages of the clinical trial recruitment and retention workflow through methods distinct from conventional approaches. For example, the study by Gligorijevic *et al*. (2019) [105] developed a system to rank clinical investigators according to their projected enrollment performance, boosting recruitment efficiency by matching eligible investigators instead of participants. The study by Beaulieu *et al*. [100] used structured clinical trial datasets from PRO-ACT and Answer ALS to evaluate a net penalized Cox proportional hazards model, which was developed for survival prediction for neurodegenerative diseases and the potential of trial admission. Overall, these seven AI approaches introduced novel approaches for streamlining clinical trial processes and demonstrated the potential to enhance recruitment effectiveness.

#### Digital tools

Digital tools were also applied to facilitate recruitment and retention workflows. For example, the chatbot-based eligibility assistants developed by Chuan *et al*. [82] demonstrated impressive performance (over 94% accuracy) and greater perceived usability and interactivity in addressing patients’ needs compared to traditional websites. Telemedicine platforms developed by Hardy–Abeloos *et al*. [84] conducted descriptive and comparative analyses for telemedicine’s impact on clinical trial enrollment odds, offering an alternative option to in-person visits, and the study indicated no significant difference in patient enrollment between Telemedicine and in-person consultations. Another study by Do *et al*. [85] built the MPACT platform for the Veterans Health Administration, integrating structured data from the Corporate Data Warehouse, unstructured clinical notes via NLP, and real-time patient schedules, resulting in a considerable reduction in patient data processing time (from 6 hours to under 1 hour). These studies collectively emphasized the potential of digital tools to increase the efficiency of clinical trial operations.

#### Offering clinical trial information

Providing additional clinical trial information to potential patient candidates has enhanced both clinical trial recruitment and retention. For instance, in Borno et al.’s study, the online platform Trial Library used ePath to enhance underrepresented patients’ interest in participating in cancer trials; a notable percentage of contacted patients completed trial surveys and were enrolled. In a study by Ma *et al*. [110], the EHR notification system for patient-provider communication increased recruitment by 10.1% through automatic notifications and referrals. Furthermore, AI-driven tools and educational platforms aimed at improving clinical trial literacy could lead to positive shifts in patient attitudes, including a greater willingness to participate in future trials.

#### Increase adherence and reduce loss

AI tools designed to improve adherence in clinical trials and minimize unwanted loss of follow-up are also essential tools that could enhance clinical trial retention. For example, a recommender system developed by Chen *et al*. [86] for smoking cessation and telehealth/video visits has been associated with an improved retention rate of between 60% and 85%. A real-time text processing system built by Delozier *et al*. [95] could reduce the time required for drug events and study consent, cutting down follow-up time by 35% by simplifying the procedures between adverse events and patient consent. With the alert system in place, the monthly rate of diTdP enrollment was 4.15 times higher compared to traditional recruitment methods. Furthermore, a remote patient monitoring platform designed by Shafner *et al*. [96] in a 2018 schizophrenia trial demonstrated improved adherence rates, with non-adherence ranging from 8.3% to 10.4% – a significant reduction from the 39%–50% non-adherence rates observed in clinical trials and real-world settings. Collectively, these examples illustrate the potential of AI tools in boosting clinical trial retention.

### Heterogeneity assessment

Heterogeneity is substantial across all task-metric strata, based on Table 2. The *I* ^2^ is consistently over 90%, indicating that most of the variability reflected real between-study differences rather than sampling error. There was dispersion expected; thus, the pooled means should be carefully read as descriptive summaries. Several strata have small k, which might inflate the heterogeneity; however, the persistently high *I* ^2^ and *τ* ^2^, even where k is large, confirm the between-study heterogeneity. Possible underlying reasons for the large between-study heterogeneity include differences in task definitions regarding different clinical trials, different underlying data sources, variation in model families/training, and the study design. Full details can be found in the Supplementary Material 2 (*Appendix Table 1.heterogeneity assessment*).

**Table 2.** Between-study heterogeneity by task and metric: for each task–metric stratum, the number of studies (*k*), *τ* ^2^ (between-study variance), and *I* ^2^ (% variability due to heterogeneity) were reported. Consistently high *I* ^2^ indicates substantial cross-study dispersion in reported performance

Task	Outcome metric	Number of studies (k)	*τ* ^2^ (between-study variance) [1]	*I* ^2^ (%)^*^
Classify EC	Accuracy	11	1.068	99.984
	F1-score	13	1.251	99.979
	Precision	14	1.053	99.974
	Sensitivity	12	1.701	99.602
Identify EC	Accuracy	12	0.499	94.489
	F1-score	33	0.248	91.163
	Precision	31	0.580	95.930
	Sensitivity	32	0.392	94.697
Identify patients	AUC	8	0.741	99.990
	Accuracy	13	0.357	98.650
	F1-score	6	3.674	99.082
	Precision	19	1.764	99.960
	Sensitivity	19	2.925	99.930
	Specificity	12	1.692	99.464
Screen patients	AUC	9	0.265	92.098
	Accuracy	2	0.442	90.973
	F1-score	6	0.290	82.252
	Precision	5	10.563	99.634
	Sensitivity	11	0.747	95.768
	Specificity	4	3.112	99.264

* *τ* ^2^ and *I* ^2^ are rounded to 3 decimal places.

### Discussion

Our analysis evaluated various AI tools used in clinical trial recruitment and retention, including patient screening, eligibility criteria extraction, and cohort identification. Overall, AI tools showed strong potential to enhance efficiency and reduce manual workload and costs. Studies reported high accuracy in pre-screening and exclusion of ineligible patients, reliable precision in extracting and defining eligibility criteria, and strong performance in classifying criteria with high sensitivity, precision, and accuracy. Cohort and site identification showed moderate discriminative performance, highlighting areas for further model refinement.

#### Limited coverage of preprint repositories

An important limitation of our search strategy is the lack of explicit querying of preprint-specific databases, such as arXiv, medRxiv, and OpenAlex. Even though our study covered an extensive scope of peer-reviewed studies, they didn’t reflect a systematic or exhaustive search of the broader preprint landscape. As preprints are increasingly relevant in fast-moving fields such as AI in healthcare, future reviews may benefit from incorporating dedicated preprint sources to ensure more comprehensive coverage.

#### Different focus of different models

Throughout all clinical workflow stages and performance metrics, the observed variability in plots was not solely determined by model reliability but also reflected differing model priorities. For example, some models were designed to avoid missing critical information, while others focused on minimizing the risk of enrolling ineligible patients. This divergence in prioritizing metrics led to a wide range in certain reported values (e.g., the specificity of screening patients varied from 0.46 to 0.95).

#### Heterogeneity of sample size

In our meta-analysis, sample sizes varied significantly across four categories. In the patient screening category, the number of patients evaluated ranged from fewer than 100 to tens of thousands. Similarly, in the patient identification category, datasets ranged from as few as 86 patients to as many as 72 million. In the eligibility criteria category, there was a notable heterogeneity in sample size, although it was less severe compared to the screening and identification categories, with a minimum of 86 annotated records and a maximum of 1600 entities. In the eligibility criteria classification category, sample sizes ranged from as small as 54 annotations to as large as 600,000 text-based samples, indicating significant variability.

Two primary factors likely contributed to this heterogeneity. First, the study design and the target disease can significantly influence variations in sample size. For example, under patient screening studies, Widera *et al*. [53] had a sample size of 74 patients and focused on the progression of knee osteoarthritis, a more narrowly defined condition, while Shi *et al*. [113] evaluated nine different diseases, with a sample size of 86,292 patients. Second, data access could also be a potential reason for the sample size heterogeneity. For example, in the 2019 study by Chen *et al*. [114], the investigators were assigned only 86 pre-annotated patient records, whereas investigators in the 2023 study by Hassan *et al*. [115] had access to an entire structured EHR patient record. The large variability in sample size across models might affect the pooled outcome of the meta-analysis, and the estimates might not represent the underlying true effect, leading to reduced statistical power in the results.

#### Insufficient report of model performance

Most (*n* = 104, 83.2%) of the papers did not report all 5 metrics (sensitivity, specificity, precision, F1-score, and AUC) in the included studies. The details on how many models reported each performance matrix can be found in Supplementary Material 1 (*Appendix D: Others of the Appendix.additional content*). This inconsistency in reporting hindered the ability to generate comprehensive pooled estimates across all performance dimensions. Furthermore, some studies did not provide explicit sample sizes, which further diminished the statistical power and limited the robustness of pooling. Future research would benefit from integrating the existing guidelines [116,117] with an additional section for standardized performance reporting to ensure that all relevant metrics and sample sizes are consistently documented.

#### Fairness concerns

Only a small portion (16.8%, *n* = 21) of the studies in our review directly addressed concerns about bias. For example, models designed specifically to address disparities in transgender recruitment and the recommendation to exercise caution regarding the potential introduction of bias and decreased generalizability when utilizing synthetic cohorts in leukemia studies [67,114]. The details of the papers discussing bias have been documented in the Supplementary Material 5 (*Appendix Table 4.categorization*). Though rarely discussed, bias in AI tools for recruitment and retention is evident and stems from data and algorithmic processes. Unchecked, it risks being perpetuated. Addressing fairness is vital for credibility and progress in clinical trials.

#### Publication bias

Publication-bias signals were present in several task–metric pairs. Full details in the Supplementary Material 6 (*Appendix Table 5.publication bias assessment*). It indicated possible small-study effects and selective reporting, thus the related unadjusted pooled performances might be inflated. Full details of funnel plots are in Supplementary Material 1 (*Appendix D: Others of the Appendix.additional content*). The results should be interpreted cautiously and future studies should prioritize larger samples and complete metric reporting.

#### Preprints and evidence inclusion

Though our search strategy did not explicitly query standalone preprint repositories (e.g., arXiv, medRxiv), it is possible that a small number of preprint records were retrieved incidentally through databases we searched (e.g., PubMed Central, which has begun indexing select NIH preprints: https://pmc.ncbi.nlm.nih.gov/about/nihpreprints/). In this review, we did not include preprints in the final evidence base in order to minimize the risk and focus on peer-reviewed literature. Readers who wish to incorporate preprints can further expand the search to dedicated preprint repositories and then re-run the same screening workflow.

## Supporting information

10.1017/cts.2026.10743.sm001 Yin et al. supplementary material 1 Yin et al. supplementary material

10.1017/cts.2026.10743.sm002 Yin et al. supplementary material 2 Yin et al. supplementary material

10.1017/cts.2026.10743.sm003 Yin et al. supplementary material 3 Yin et al. supplementary material

10.1017/cts.2026.10743.sm004 Yin et al. supplementary material 4 Yin et al. supplementary material

10.1017/cts.2026.10743.sm005 Yin et al. supplementary material 5 Yin et al. supplementary material

10.1017/cts.2026.10743.sm006 Yin et al. supplementary material 6 Yin et al. supplementary material

## Supplementary material

The supplementary material for this article can be found at https://doi.org/10.1017/cts.2026.10743.
