# Supplementary material for: Artificial intelligence in clinical trial participant recruitment and retention: A scoping review and meta-analysis
Source: J Clin Transl Sci. 2026 Apr 23;10(1):e98. doi: 10.1017/cts.2026.10743 (PMC13312366; doi:10.1017/cts.2026.10743)
Supplement: Yin et al. supplementary material 1 — Yin et al. supplementary material [file S2059866126107432sup001.pdf]

Appendix A: Extra Details of Methods

A comprehensive scoping review was conducted to investigate the use of AI technologies in enhancing recruitment and retention in clinical trials. This review adhered to the Joanna Briggs Institute (JBI) methodological framework and was reported in accordance with the Preferred Reporting Items for Systematic Reviews and Scoping Reviews (PRISMA-ScR) guidelines to ensure transparency and compliance with reporting standards. The paper selection process was depicted using a PRISMA-ScR flow diagram.

Stage 1: Identifying the Research Question

The primary aim of this review was to characterize the current landscape of AI applications in clinical trial participant recruitment and retention and to explore potential biases of AI algorithms. Specifically, we addressed the following inquiries:

- What are the applications of AI in recruiting and retaining participants in clinical trials?
- What is the effectiveness of AI-based solutions?
- How do AI approaches support equitable and comprehensive participant selection?
- What gaps and potential biases arise when AI technologies are used for recruitment and retention?

These objectives guided the identification of AI use patterns across clinical trial studies, emphasizing emerging topics requiring further investigation, and enabled a concise summary of prior research.

Stage 2: Defining Inclusion Criteria

We defined the eligibility criteria to guarantee that the chosen studies include precise information relevant to our research inquiry. The development of criteria adhered to the scoping review approach established by the JBI, which outlines the types of participants, concepts, and context of the study.

We defined eligibility criteria to guarantee that included studies provided information relevant to our research inquiries. The criteria development adhered to the scoping review approach from JBI, which outlines the types of participants, concepts, and context of the study.

Overall Inclusion Criteria

To be included in this scoping review, studies must meet the following criteria:

- The study must focus on the application of AI technology.
- The study must specifically address the use of AI technology in assisting or facilitating recruitment and retention in clinical trials.

Types of participants

The scoping review aimed to gather all relevant research that demonstrated the use of AI in the recruitment and retention processes of clinical trials. We included studies involving

participants of various ages and other qualifying criteria who participated in clinical trials or retrospective/prospective research where AI was used in recruitment and retention. We also detailed important characteristics of these participants, such as age and other relevant criteria.

Context and Concept
The overarching concept of this scoping review was the use of AI in enhancing clinical trial processes, specifically focusing on recruitment and retention. This included examining the types of AI tools used, their application domains, and their effectiveness in improving trial outcomes. Further elements of interest related to AI performance in identifying patients included area under the receiver operating characteristic curve (AUC), sensitivity, specificity, positive predictive value, and negative predictive value. The review also explored outcomes related to AI applications, including their impact on trial success and potential biases.

The context of this scoping review included diverse geographic locations and healthcare settings where AI was used in clinical trials. The review also considered different cultural and social factors that influenced the use and effectiveness of AI tools, with an emphasis on understanding how these tools performed in different contexts and identifying any disparities or biases in their application.

Types of evidence sources
We incorporated both published and unpublished papers that covered a range of study types. These included analytical observational studies such as prospective and retrospective cohort studies, case-control studies, and analytical cross-sectional studies. Additionally, we included descriptive observational study designs, such as descriptive cross-sectional studies. Furthermore, our scoping review considered all individuals who participated in clinical trials or retrospective/prospective research involving the use of AI for recruitment and retention. While our primary focus was on observational studies, we also considered relevant experimental studies, reviews, and meta-analyses if they provided valuable insights into the use of AI in these contexts. We did not apply restrictions by article type; however, articles that specifically used AI as an intervention were not included.

Stage 3: Developing the Search Strategy
To identify relevant studies, we developed comprehensive search strategies tailored to each database, with the help of an experienced librarian. The search strategy used the Boolean operator OR to combine keywords and the operator AND to filter papers covering both AI and trial participant terminologies/keywords (see *Appendix B: Search Strategy* for details). Comprehensive searches utilizing both controlled vocabulary and keywords were conducted in Medline (Ovid), Cochrane Library, Embase (Elsevier), CINAHL Complete (EBSCOhost), and Scopus (Elsevier). Noncomprehensive searches were done in ACM and IEEE due to the search limitations of those databases. Only publications in English were included for feasibility. The literature search was limited to publications from 2018 to the present, corresponding to the emergence of novel AI tools and techniques. The search strategy was transformed to match different formats across databases. An experienced librarian assisted in strategy structuring, transforming, and implementing throughout the process, guaranteeing

robust searching and comprehensive identification of relevant studies. The details of our search strategy included:

- AI terms: our search strategy employed a comprehensive range of AI-related terms to ensure an inclusive review of the literature. The strategy included various AI and machine learning methodologies, such as deep learning and neural networks, as well as specific applications like natural language processing and generative adversarial networks. We also incorporated both broad and specific terms related to AI tools and techniques, ensuring that our search captured all studies using AI for recruitment and retention in clinical trials.
- Trial Participation Terms: our search strategy aimed to capture all relevant clinical trial research related to recruitment and retention while excluding irrelevant papers. It included terms related to trial participation, such as patient selection, research subjects, and participant engagement. It also incorporated recruitment and retention terminologies like eligibility determination, participant matching, prescreen, dropout, and follow-up.
- Loose trial participation terms in title: our search strategy also included highly relevant keywords specifically targeted in titles. These included recruitment- and retention-stage-specific terminologies, random trial/trials, clinical trial/trials, etc.
- Restriction on publication time: our search strategy included only papers published from 2018 to the present.
- Restriction on publication type: our search strategy excluded editorials, letters, and comments.
- Restriction on language: our search strategy included only papers published in English.
- No constraint on the type of clinical trial or source countries of the papers.

This extensive search strategy aimed to gather diverse references, ensuring the inclusion of the latest advancements and applications of AI in the clinical trial recruitment and retention domain.

Stage 4: Paper Screening and Source of Evidence Selection

The source selection process for this scoping review followed a structured approach to ensure comprehensive and unbiased inclusion of relevant evidence. This process included title and abstract screening followed by full-text screening, with all stages performed by at least two independent reviewers. Covidence was employed to identify and remove all duplicate articles. Every publication that met our inclusion criteria was subjected to title and abstract screening. In instances of disagreement or discrepancy, consensus was achieved through group discussions by involving an additional third reviewer. The consistency and agreement among reviewers were evaluated using a training set of 100 papers. During the training, each paper was reviewed by two reviewers, and any disagreements were discussed and resolved.

Supplementary Methods: Large Language Model–Assisted Abstract Screening

To support efficiency and consistency during initial screening, we incorporated a large language model, GPT-4o mini, by calling the ChatGPT API, to generate relevance scores for

each paper retrieved from the database search. We provided the model with predefined inclusion criteria (see *Defining Inclusion Criteria* section for details). For each title and abstract pair, the model generated a numerical relevance score ranging from 0 to 100, where higher values indicated stronger predicted alignment with the inclusion criteria. These scores were subsequently used to rank records and guide the order of manual review during the screening process. All exclusion decisions were made by human reviewers.

The exact prompt used was:
“We are trying to conduct a PRISMA systematic review study titled ‘AI for clinical trial recruitment’. You will take the role of a reviewer to check the abstracts and titles if they should be selected for further assessment and full-text screening. Please give a score between 1–100 with no explanation for the following abstract and title on how likely this article should be included, where 70 and above means we should screen the paper and 69 and below means we should not screen the paper.
Our inclusion criteria are the following:
Criterion A: Study has to have implemented some form of artificial intelligence, machine learning, natural language processing, or computer vision.
Criterion B: Study has to be related to a clinical trial.
Criterion C: Study has to be related to participant recruitment, enrolment, selection, engagement, or retention.”

Records were ranked from highest to lowest score to guide the order in which human reviewers screened abstracts. Based on pilot testing with a manually evaluated test set, papers scoring >58 were classified as “likely to meet” inclusion criteria (n = 1,236). Papers scoring <40 were considered “unlikely to meet” criteria (n = 12,720). Papers in the intermediate range (40–58, n = 4,785) were classified as ambiguous. The thresholds provided an estimate of the model’s confidence but were only used as guidance. All records underwent two independent reviewers' screening regardless of score, and discrepancies were resolved by consensus with a third reviewer. This hybrid approach reduced manual workload while ensuring that final decisions were made effectively and accurately.

Appendix B: Search Strategy

Steph Hendren, MLIS; Duke University Medical Center Library, Duke University School of Medicine

Date of completed search: June 28, 2024

Database: Medline (via Ovid)

Set #	Search Strategy	Results
1<br>AI terms	exp artificial intelligence/ OR exp deep learning/ OR exp image interpretation, computer-assisted/ OR exp machine learning/ OR exp neural networks, computer/ OR exp supervised machine learning/ OR exp support vector machine/ OR exp unsupervised machine learning/ OR (AI OR NLP OR BERT OR chatgpt OR copilot OR cnn OR "deep learning" OR "generative adversarial network" OR "generative adversarial networks" OR GAN OR GANs OR "kalman filter" OR "language model" OR "language models" OR LLM OR LLMs OR machine-learning OR "neural network" OR "neural networks" OR "neural net" OR "neural nets" OR openai OR "sentiment analysis" OR "named entity recognition").ti,ab. OR ((artificial OR artificially OR augmented OR augmenting OR automated OR automating OR machine OR computer OR computers OR cognitive) adj (intelligence OR computation OR computations OR computing OR reasoning)).ti,ab. OR ((computer OR computers OR machine OR machines OR "natural language") adj (assisted OR assisting OR assists OR processing OR processed OR processor OR processors OR learning OR learn OR learns OR learned OR vision OR visions)).ti,ab. OR ((hierarchial OR deep) adj3 (learn*)).ti,ab.	1020897
2<br>trial participation terms	((exp Clinical Trials as Topic/ OR exp clinical trial/ OR exp randomized controlled trial/ OR ((random* OR clinical OR control OR controlled OR research OR prospective OR cohort OR diagnosis OR prognosis OR intervention*).ti,ab. AND (trial OR trials).ti,ab.)) AND (exp Patient Selection/ OR exp Research Subjects/ OR (participant OR participants OR patient OR patients OR patients' OR patient's OR "research subject" OR "research subjects").ti,ab.) AND (exp Eligibility Determination/ OR (participat* OR engag* OR involv* OR enroll* OR retention OR retain* OR eligibility OR eligible OR select* OR prescreen* OR matching OR matched OR matches OR match OR follow-up OR "follow up" OR "followed up" OR "following up" OR dropout OR dropouts OR "drop out" OR "drop outs" OR "dropping out" OR "dropped out" OR attrition).ti,ab.))	601159

3<br><br>trial participation terms	(exp Clinical Trials as Topic/ OR exp Patient Selection/ OR exp Research Subjects/) AND (exp Eligibility Determination/ OR (participat* OR engag* OR involv* OR enroll* OR retention OR retain* OR eligibility OR eligible OR select* OR prescreen* OR matching OR matched OR matches OR match OR follow-up OR "follow up" OR "followed up" OR "following up" OR dropout OR dropouts OR "drop out" OR "drop outs" OR "dropping out" OR "dropped out" OR attrition).ti.)	19187
4<br><br>trial participation terms	((((random* OR clinical OR control OR controlled OR research OR prospective OR cohort OR diagnosis OR prognosis OR intervention*).ti. AND (trial OR trials).ti.) OR (participant OR participants OR patient OR patients OR patients' OR patient's OR "research subject" OR "research subjects").ti.) AND (participat* OR engag* OR involv* OR enroll* OR retention OR retain* OR eligibility OR eligible OR select* OR prescreen* OR matching OR matched OR matches OR match OR follow-up OR "follow up" OR "followed up" OR "following up" OR dropout OR dropouts OR "drop out" OR "drop outs" OR "dropping out" OR "dropped out" OR attrition).ti.)	88200
5	2 OR 3 OR 4	675482
6	1 AND 5	21141
7	Date filter: 2018 to present	7946
8	7 NOT (Editorial OR Letter OR Comment).pt.	7848
9	limit 8 to English	7764

Database: Embase (via Elsevier)

Note: all searches were run in "results" tab

Set #	Search Strategy	Results
1	'artificial intelligence'/exp OR 'machine learning'/exp OR 'artificial neural network'/exp OR 'deep learning'/exp OR (AI OR NLP OR BERT	704636

AI terms	OR chatgpt OR copilot OR cnn OR 'deep learning' OR 'generative adversarial network' OR 'generative adversarial networks' OR GAN OR GANs OR 'kalman filter' OR 'language model' OR 'language models' OR LLM OR LLMs OR machine-learning OR 'neural network' OR 'neural networks' OR 'neural net' OR 'neural nets' OR openai OR 'sentiment analysis' OR 'named entity recognition'):ti,ab OR ((artificial OR artificially OR augmented OR augmenting OR automated OR automating OR machine OR computer OR computers OR cognitive) NEAR/1 (intelligence OR computation OR computations OR computing OR reasoning)):ti,ab OR ((computer OR computers OR machine OR machines OR 'natural language') NEAR/1 (assisted OR assisting OR assists OR processing OR processed OR processor OR processors OR learning OR learn OR learns OR learned OR vision OR visions)):ti,ab OR ((hierarchial OR deep) NEAR/3 (learn*)):ti,ab	
2<br>trial participation terms	(('clinical trial (topic)'/exp OR 'clinical trial'/exp OR 'randomized controlled trial'/exp OR ((random* OR clinical OR control OR controlled OR research OR prospective OR cohort OR diagnosis OR prognosis OR intervention*):ti,ab AND (trial OR trials):ti,ab)) AND ('patient selection'/exp OR 'research subject'/exp OR (participant OR participants OR patient OR patients OR 'research subject' OR 'research subjects'):ti,ab) AND ('eligibility'/exp OR (participat* OR engag* OR involv* OR enroll* OR retention OR retain* OR eligibility OR eligible OR select* OR prescreen* OR matching OR matched OR matches OR match OR follow-up OR 'follow up' OR 'followed up' OR 'following up' OR dropout OR dropouts OR 'drop out' OR 'drop outs' OR 'dropping out' OR 'dropped out' OR attrition):ti,ab))	1083361
3<br>trial participation terms	('clinical trial (topic)'/exp OR 'patient selection'/exp OR 'research subject'/exp) AND ('eligibility'/exp OR (participat* OR engag* OR involv* OR enroll* OR retention OR retain* OR eligibility OR eligible OR select* OR prescreen* OR matching OR matched OR matches OR match OR follow-up OR 'follow up' OR 'followed up' OR 'following up' OR dropout OR dropouts OR 'drop out' OR 'drop outs' OR 'dropping out' OR 'dropped out' OR attrition):ti)	24935
4<br>trial participation terms	((((random* OR clinical OR control OR controlled OR research OR prospective OR cohort OR diagnosis OR prognosis OR intervention*):ti AND (trial OR trials):ti) OR (participant OR participants OR patient OR patients OR 'research subject' OR 'research subjects'):ti) AND (participat* OR engag* OR involv* OR enroll* OR retention OR retain* OR eligibility OR eligible OR select* OR	113831

	prescreen* OR matching OR matched OR matches OR match OR follow-up OR 'follow up' OR 'followed up' OR 'following up' OR dropout OR dropouts OR 'drop out' OR 'drop outs' OR 'dropping out' OR 'dropped out' OR attrition):ti)	
5	2 OR 3 OR 4	1782929
6	1 AND 5	42133
7	Date filter: 2018 to present	19095
8	7 NOT ('editorial'/exp OR [editorial]/lim OR 'letter'/exp OR [letter]/lim OR 'note'/exp OR [note]/lim OR [conference abstract]/lim OR 'conference abstract'/exp OR 'conference abstract'/it)	10566
9	limit 8 to English	10403

Database: CINAHL Complete (via EBSCOhost)

Set #	Search Strategy	Results
1<br><br>AI terms	MH "Artificial Intelligence+" OR MH "Machine Learning+" OR MH "Deep Learning" OR MH "Image Interpretation, Computer Assisted+" OR MH "Neural Networks (Computer)" OR TITLE (AI OR NLP OR BERT OR chatgpt OR copilot OR cnn OR "deep learning" OR "generative adversarial network" OR "generative adversarial networks" OR GAN OR GANs OR "kalman filter" OR "language model" OR "language models" OR LLM OR LLMs OR machine-learning OR "neural network" OR "neural networks" OR "neural net" OR "neural nets" OR openai OR "sentiment analysis" OR "named entity recognition") OR ABSTRACT (AI OR NLP OR BERT OR chatgpt OR copilot OR cnn OR "deep learning" OR "generative adversarial network" OR "generative adversarial networks" OR GAN OR GANs OR "kalman filter" OR "language model" OR "language models" OR LLM OR LLMs OR machine-learning OR "neural network" OR "neural networks" OR "neural net" OR "neural nets" OR openai OR "sentiment analysis" OR "named entity recognition") OR TITLE ((artificial OR artificially OR augmented OR augmenting OR automated OR automating OR machine OR computer OR computers OR cognitive) N1 (intelligence OR computation OR computations OR computing OR reasoning)) OR ABSTRACT ((artificial OR artificially OR augmented	187605

	OR augmenting OR automated OR automating OR machine OR computer OR computers OR cognitive) N1 (intelligence OR computation OR computations OR computing OR reasoning)) OR TITLE ((computer OR computers OR machine OR machines OR "natural language") N1 (assisted OR assisting OR assists OR processing OR processed OR processor OR processors OR learning OR learn OR learns OR learned OR vision OR visions)) OR ABSTRACT ((computer OR computers OR machine OR machines OR "natural language") N1 (assisted OR assisting OR assists OR processing OR processed OR processor OR processors OR learning OR learn OR learns OR learned OR vision OR visions)) OR TITLE ((hierarchial OR deep) N3 (learn*)) OR ABSTRACT ((hierarchial OR deep) N3 (learn*))	
2<br><br>trial participation terms	((MH "Clinical Trials+" OR MH "Randomized Controlled Trials+" OR MH "Nonrandomized Trials" OR MH "Intervention Trials" OR MH "Clinical Trial Registry" OR ((TITLE (random* OR clinical OR control OR controlled OR research OR prospective OR cohort OR diagnosis OR prognosis OR intervention*) OR ABSTRACT (random* OR clinical OR control OR controlled OR research OR prospective OR cohort OR diagnosis OR prognosis OR intervention*)) AND (TITLE (trial OR trials) OR ABSTRACT (trial OR trials)))) AND (MH "Patient Selection" OR MH "Research Subjects+" OR TITLE (participant OR participants OR patient OR patients OR patients' OR patient's OR "research subject" OR "research subjects") OR ABSTRACT (participant OR participants OR patient OR patients OR patients' OR patient's OR "research subject" OR "research subjects")) AND (MH "Eligibility Determination" OR TITLE (participat* OR engag* OR involv* OR enroll* OR retention OR retain* OR eligibility OR eligible OR select* OR prescreen* OR matching OR matched OR matches OR match OR follow-up OR "follow up" OR "followed up" OR "following up" OR dropout OR dropouts OR "drop out" OR "drop outs" OR "dropping out" OR "dropped out" OR attrition) OR ABSTRACT (participat* OR engag* OR involv* OR enroll* OR retention OR retain* OR eligibility OR eligible OR select* OR prescreen* OR matching OR matched OR matches OR match OR follow-up OR "follow up" OR "followed up" OR "following up" OR dropout OR dropouts OR "drop out" OR "drop outs" OR "dropping out" OR "dropped out" OR attrition)))	6661

3<br><br>trial participation terms	(MH "Clinical Trials+" OR MH "Randomized Controlled Trials+" OR MH "Nonrandomized Trials" OR MH "Intervention Trials" OR MH "Clinical Trial Registry" OR exp Patient Selection/ OR exp Research Subjects/) AND (MH "Eligibility Determination" OR TITLE (participat* OR engag* OR involv* OR enroll* OR retention OR retain* OR eligibility OR eligible OR select* OR prescreen* OR matching OR matched OR matches OR match OR follow-up OR "follow up" OR "followed up" OR "following up" OR dropout OR dropouts OR "drop out" OR "drop outs" OR "dropping out" OR "dropped out" OR attrition))	799
4<br><br>trial participation terms	((TITLE (random* OR clinical OR control OR controlled OR research OR prospective OR cohort OR diagnosis OR prognosis OR intervention*) AND TITLE (trial OR trials)) OR TITLE (participant OR participants OR patient OR patients OR patients' OR patient's OR "research subject" OR "research subjects")) AND TITLE (participat* OR engag* OR involv* OR enroll* OR retention OR retain* OR eligibility OR eligible OR select* OR prescreen* OR matching OR matched OR matches OR match OR follow-up OR "follow up" OR "followed up" OR "following up" OR dropout OR dropouts OR "drop out" OR "drop outs" OR "dropping out" OR "dropped out" OR attrition)	4579
5	2 OR 3 OR 4	10022
6	1 AND 5	239
7	Date filter: 2018 to present	116
8	limit 8 to English	112

Database: Scopus (via Elsevier)

Set #	Search Strategy	Results
1<br><br>AI terms	TITLE-ABS(AI OR NLP OR BERT OR chatgpt OR copilot OR cnn OR {deep learning} OR {generative adversarial network} OR {generative adversarial networks} OR GAN OR GANs OR {kalman filter} OR {language model} OR {language models} OR LLM OR LLMs OR machine-learning OR {neural network} OR {neural	2194165

	networks} OR {neural net} OR {neural nets} OR openai OR {sentiment analysis} OR {named entity recognition}) OR TITLE-ABS((artificial OR artificially OR augmented OR augmenting OR automated OR automating OR machine OR computer OR computers OR cognitive) W/1 (intelligence OR computation OR computations OR computing OR reasoning)) OR TITLE-ABS((computer OR computers OR machine OR machines OR language) W/1 (assisted OR assisting OR assists OR processing OR processed OR processor OR processors OR learning OR learn OR learns OR learned OR vision OR visions)) OR TITLE-ABS((hierarchial OR deep) W/3 (learn*))	
2<br><br>trial participation terms	TITLE-ABS((random* OR clinical OR control OR controlled OR research OR prospective OR cohort OR diagnosis OR prognosis OR intervention*) AND (trial OR trials)) AND TITLE-ABS(participant OR participants OR patient OR patients OR patients' OR patient's OR {research subject} OR {research subjects}) AND TITLE-ABS(participat* OR engag* OR involv* OR enroll* OR retention OR retain* OR eligibility OR eligible OR select* OR prescreen* OR matching OR matched OR matches OR match OR follow-up OR {follow up} OR {followed up} OR {following up} OR dropout OR dropouts OR {drop out} OR {drop outs} OR {dropping out} OR {dropped out} OR attrition)	22508
3<br><br>trial participation terms	TITLE((((random* OR clinical OR control OR controlled OR research OR prospective OR cohort OR diagnosis OR prognosis OR intervention*) AND (trial OR trials)) OR (participant OR participants OR patient OR patients OR patients' OR patient's OR {research subject} OR {research subjects}) AND (participat* OR engag* OR involv* OR enroll* OR retention OR retain* OR eligibility OR eligible OR select* OR prescreen* OR matching OR matched OR matches OR match OR follow-up OR {follow up} OR {followed up} OR {following up} OR dropout OR dropouts OR {drop out} OR {drop outs} OR {dropping out} OR {dropped out} OR attrition)))	15063
4	2 OR 3	37004
6	1 AND 4	382
5	Date filter: 2018 to present	279

8	Document type selected: Article	218
9	limit 8 to English	208

Database: Cochrane Central (via WileyOnline)

Note: search was conducted in "search manager" tab

Set #	Search Strategy	Results
1<br><br>AI terms	[mh "artificial intelligence"] OR [mh "deep learning"] OR [mh "image interpretation, computer-assisted"] OR [mh "machine learning"] OR [mh "neural networks, computer"] OR [mh "supervised machine learning"] OR [mh "support vector machine"] OR [mh "unsupervised machine learning"] OR (AI:ti,ab OR NLP:ti,ab OR BERT:ti,ab OR chatgpt:ti,ab OR copilot:ti,ab OR cnn:ti,ab OR "deep learning":ti,ab OR "generative adversarial network":ti,ab OR "generative adversarial networks":ti,ab OR GAN:ti,ab OR GANs:ti,ab OR "kalman filter":ti,ab OR "language model":ti,ab OR "language models":ti,ab OR LLM:ti,ab OR LLMs:ti,ab OR machine-learning:ti,ab OR "neural network":ti,ab OR "neural networks":ti,ab OR "neural net":ti,ab OR "neural nets":ti,ab OR openai:ti,ab OR "sentiment analysis":ti,ab OR "named entity recognition":ti,ab) OR ((artificial:ti,ab OR artificially:ti,ab OR augmented:ti,ab OR augmenting:ti,ab OR automated:ti,ab OR automating:ti,ab OR machine:ti,ab OR computer:ti,ab OR computers:ti,ab OR cognitive:ti,ab) NEXT (intelligence:ti,ab OR computation:ti,ab OR computations:ti,ab OR computing:ti,ab OR reasoning:ti,ab)) OR ((computer:ti,ab OR computers:ti,ab OR machine:ti,ab OR machines:ti,ab OR "natural language":ti,ab) NEXT (assisted:ti,ab OR assisting:ti,ab OR assists:ti,ab OR processing:ti,ab OR processed:ti,ab OR processor:ti,ab OR processors:ti,ab OR learning:ti,ab OR learn:ti,ab OR learns:ti,ab OR learned:ti,ab OR vision:ti,ab OR visions:ti,ab)) OR ((hierarchial:ti,ab OR deep:ti,ab) NEAR/3 (learn*:ti,ab))	27435

2<br><br>trial participation terms	(([mh "Clinical Trials as Topic"] OR [mh "clinical trial"] OR [mh "randomized controlled trial"] OR ((random*:ti,ab OR clinical:ti,ab OR control:ti,ab OR controlled:ti,ab OR research:ti,ab OR prospective:ti,ab OR cohort:ti,ab OR diagnosis:ti,ab OR prognosis:ti,ab OR intervention*:ti,ab) AND (trial:ti,ab OR trials:ti,ab))) AND ([mh "Patient Selection"] OR [mh "Research Subjects"] OR (participant:ti,ab OR participants:ti,ab OR patient:ti,ab OR patients:ti,ab OR patients':ti,ab OR patient's:ti,ab OR "research subject":ti,ab OR "research subjects":ti,ab)) AND ([mh "Eligibility Determination"] OR (participat*:ti,ab OR engag*:ti,ab OR involv*:ti,ab OR enroll*:ti,ab OR retention:ti,ab OR retain*:ti,ab OR eligibility:ti,ab OR eligible:ti,ab OR select*:ti,ab OR prescreen*:ti,ab OR matching:ti,ab OR matched:ti,ab OR matches:ti,ab OR match:ti,ab OR follow-up:ti,ab OR "follow up":ti,ab OR "followed up":ti,ab OR "following up":ti,ab OR dropout:ti,ab OR dropouts:ti,ab OR "drop out":ti,ab OR "drop outs":ti,ab OR "dropping out":ti,ab OR "dropped out":ti,ab OR attrition:ti,ab)))	363752
3<br><br>trial participation terms	([mh "Clinical Trials as Topic"] OR [mh "Patient Selection"] OR [mh "Research Subjects"]) AND ([mh "Eligibility Determination"] OR (participat*:ti OR engag*:ti OR involv*:ti OR enroll*:ti OR retention:ti OR retain*:ti OR eligibility:ti OR eligible:ti OR select*:ti OR prescreen*:ti OR matching:ti OR matched:ti OR matches:ti OR match:ti OR follow-up:ti OR "follow up":ti OR "followed up":ti OR "following up":ti OR dropout:ti OR dropouts:ti OR "drop out":ti OR "drop outs":ti OR "dropping out":ti OR "dropped out":ti OR attrition:ti))	3381
4<br><br>trial participation terms	((((random*:ti OR clinical:ti OR control:ti OR controlled:ti OR research:ti OR prospective:ti OR cohort:ti OR diagnosis:ti OR prognosis:ti OR intervention*:ti) AND (trial:ti OR trials:ti)) OR (participant:ti OR participants:ti OR patient:ti OR patients:ti OR patients':ti OR patient's:ti OR "research subject":ti OR "research subjects":ti)) AND (participat*:ti OR engag*:ti OR involv*:ti OR enroll*:ti OR retention:ti OR retain*:ti OR eligibility:ti OR eligible:ti OR select*:ti OR prescreen*:ti OR matching:ti OR matched:ti OR matches:ti OR match:ti OR follow-up:ti OR "follow up":ti OR "followed up":ti OR "following up":ti OR dropout:ti OR dropouts:ti OR "drop out":ti OR "drop outs":ti OR "dropping out":ti OR "dropped out":ti OR attrition:ti))	24777
5	2 OR 3 OR 4	374321

6	1 AND 5	5732
7	Date filter: 2018 to present<br><br>-- 28 SRs and 3058 clinical trials	3086

Appendix C: Additional Figures

Figure 1. Sensitivity Meta-analysis for Papers Using Machine Learning for Patients Screening

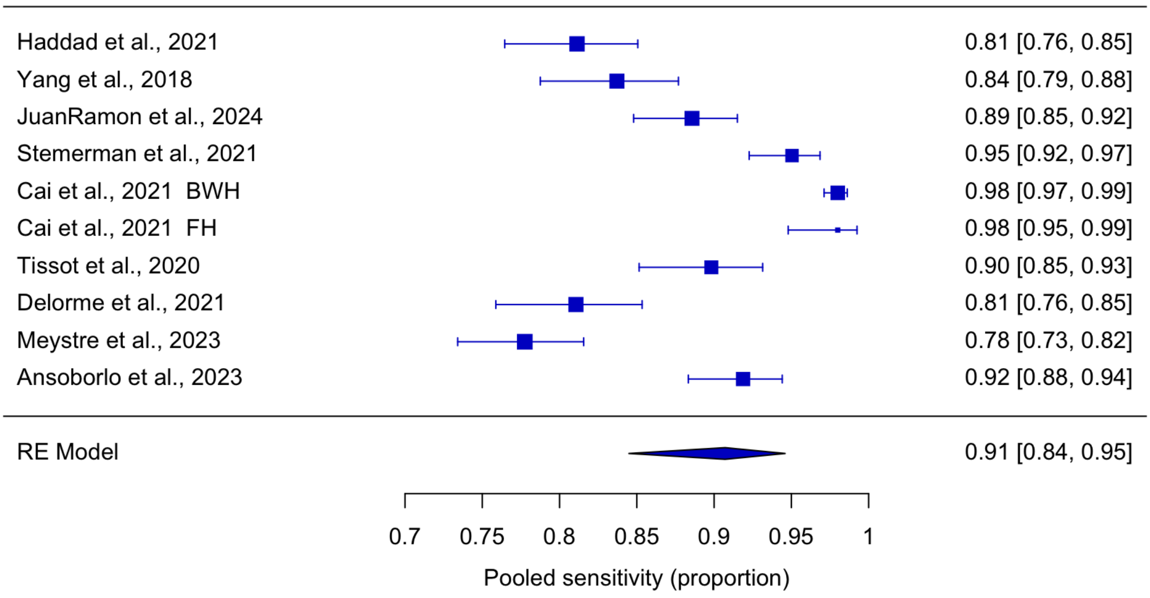

Figure 2. Specificity Meta-analysis for Papers Using Machine Learning for Patient Screening

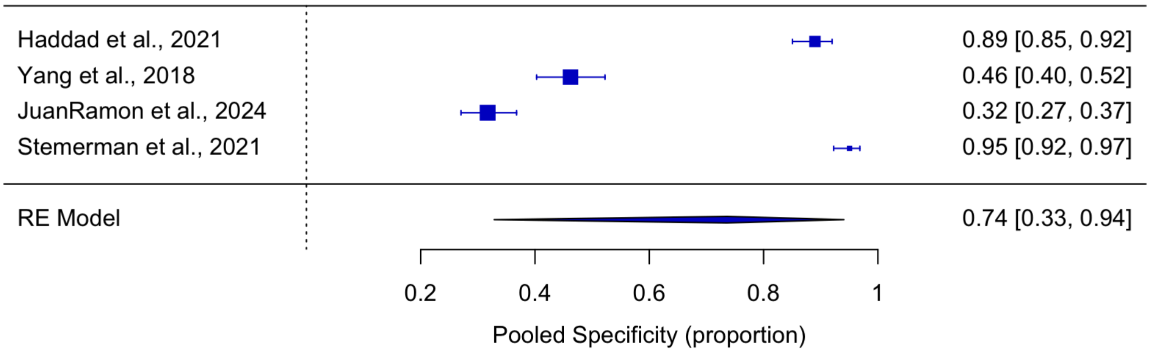

Figure 3. Precision Meta-analysis for Papers Using Machine Learning for Patient Screening

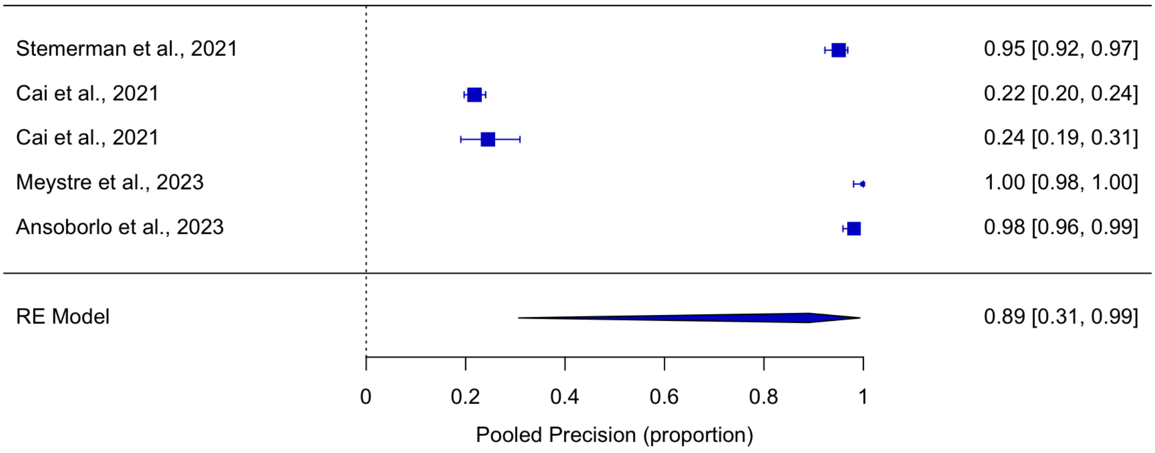

Figure 4. Accuracy Meta-analysis for Papers Using Machine Learning for Patient Screening

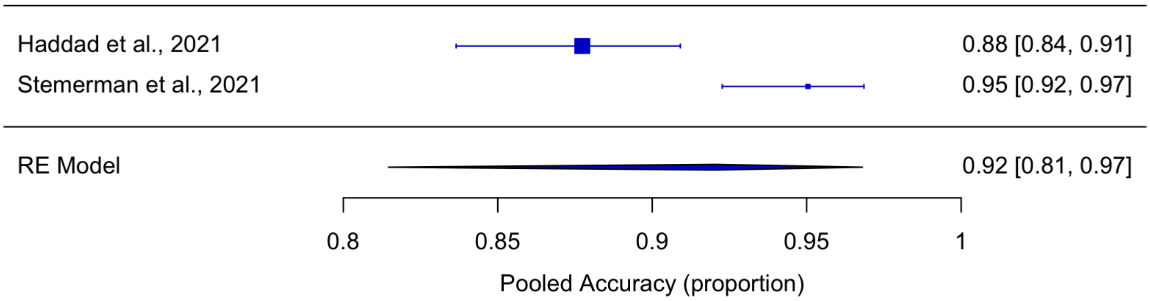

Figure 5. AUC Meta-analysis for Papers Using Machine Learning for Patient Screening

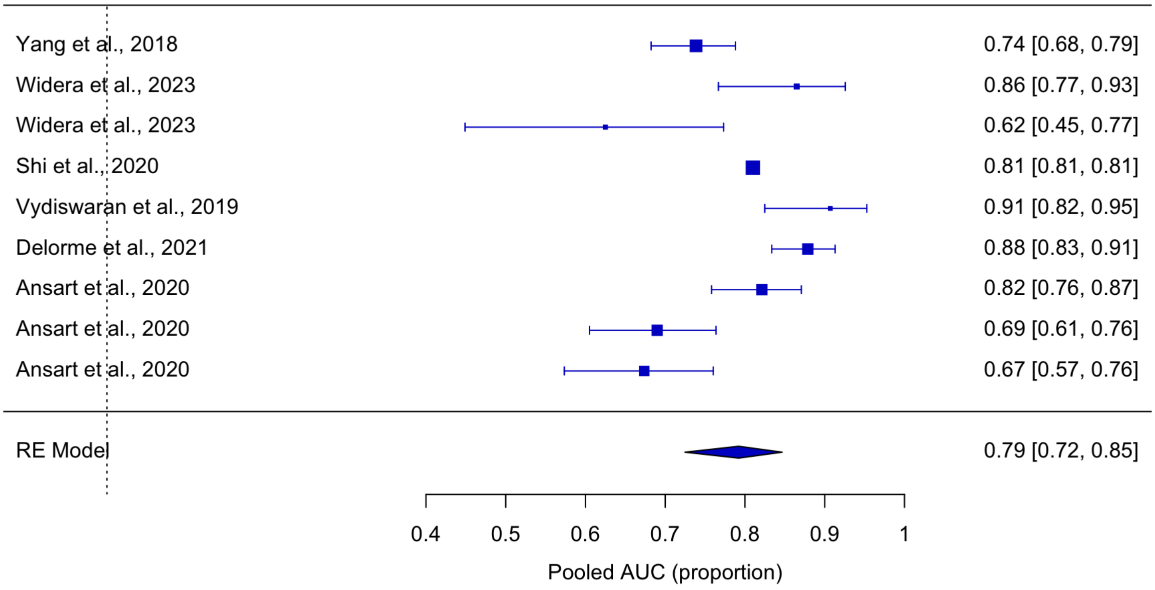

Figure 6. F-1 Score Meta-analysis for Papers Using Machine Learning for Patient Screening

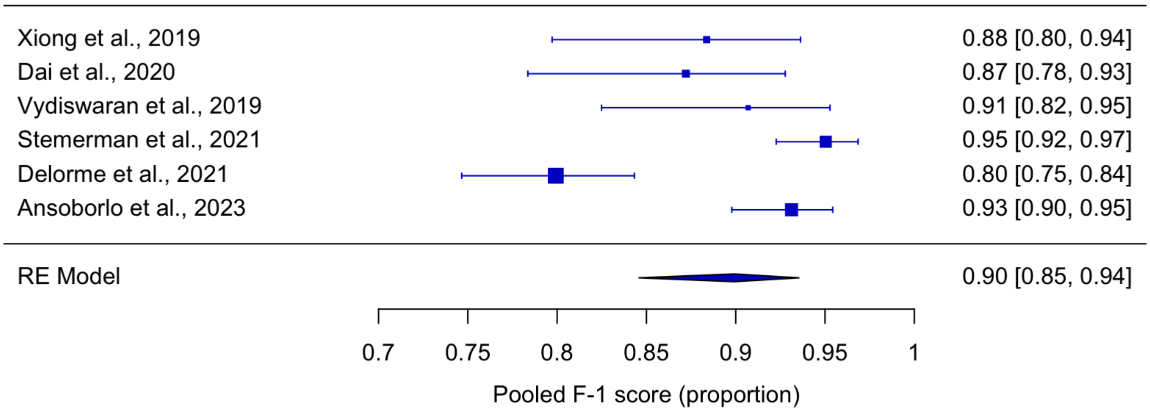

Figure 7. Sensitivity Meta-analysis for Papers Using Machine Learning to Identify Eligibility Criteria

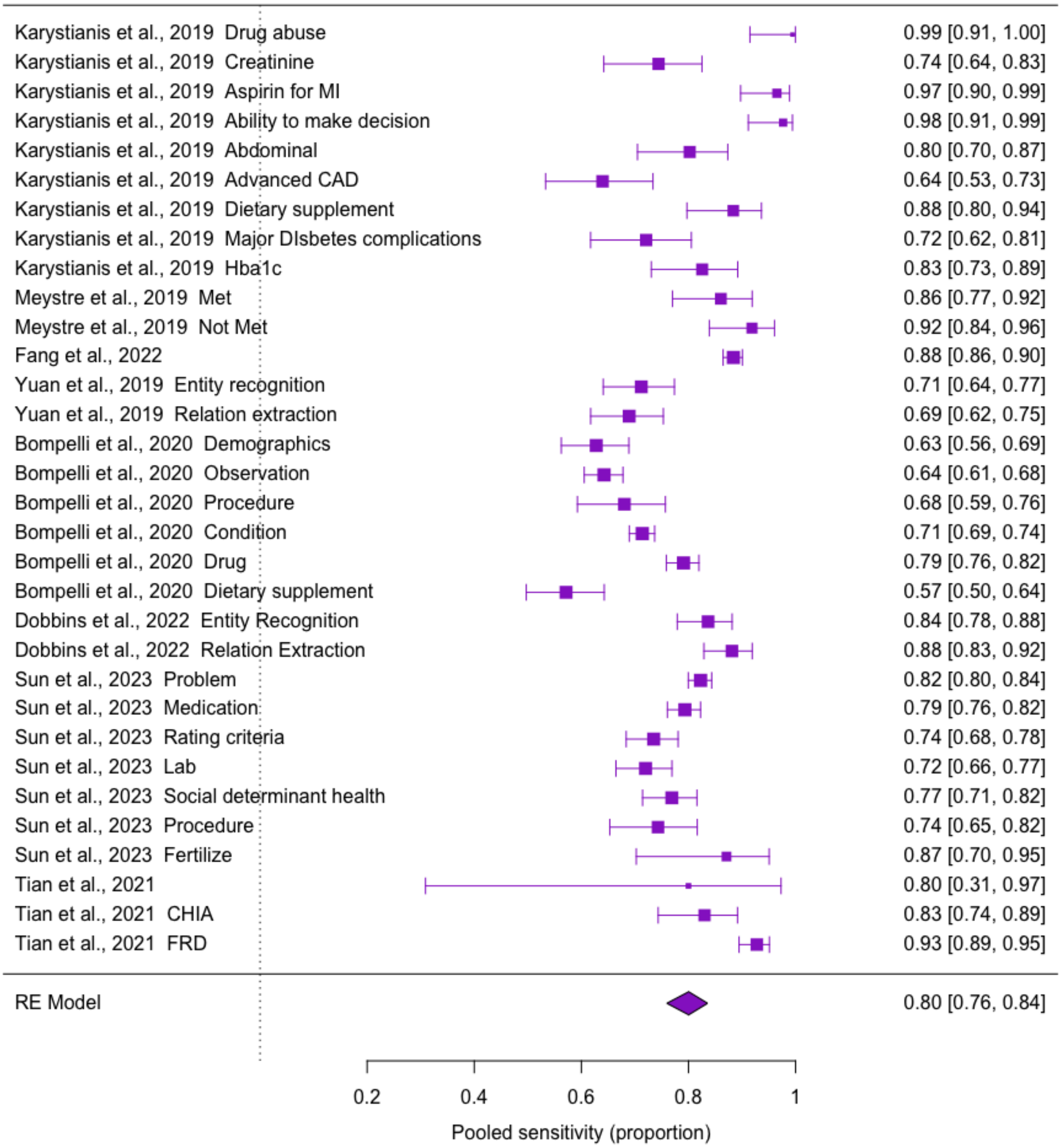

Figure 8. Precision Meta-analysis for Papers Using Machine Learning to Identify Eligibility Criteria

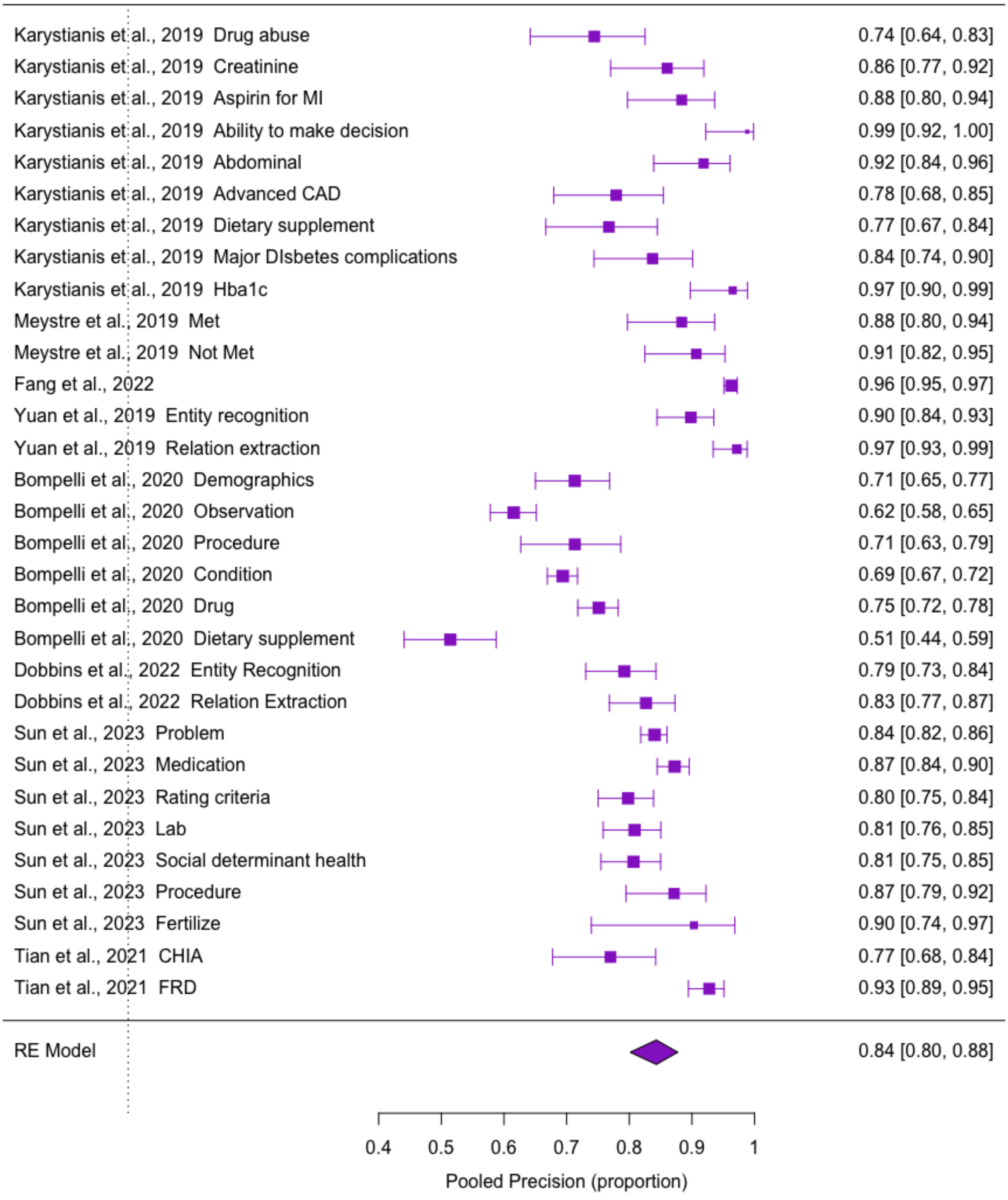

Figure 9. Accuracy Meta-analysis for Papers Using Machine Learning to Identify Eligibility Criteria

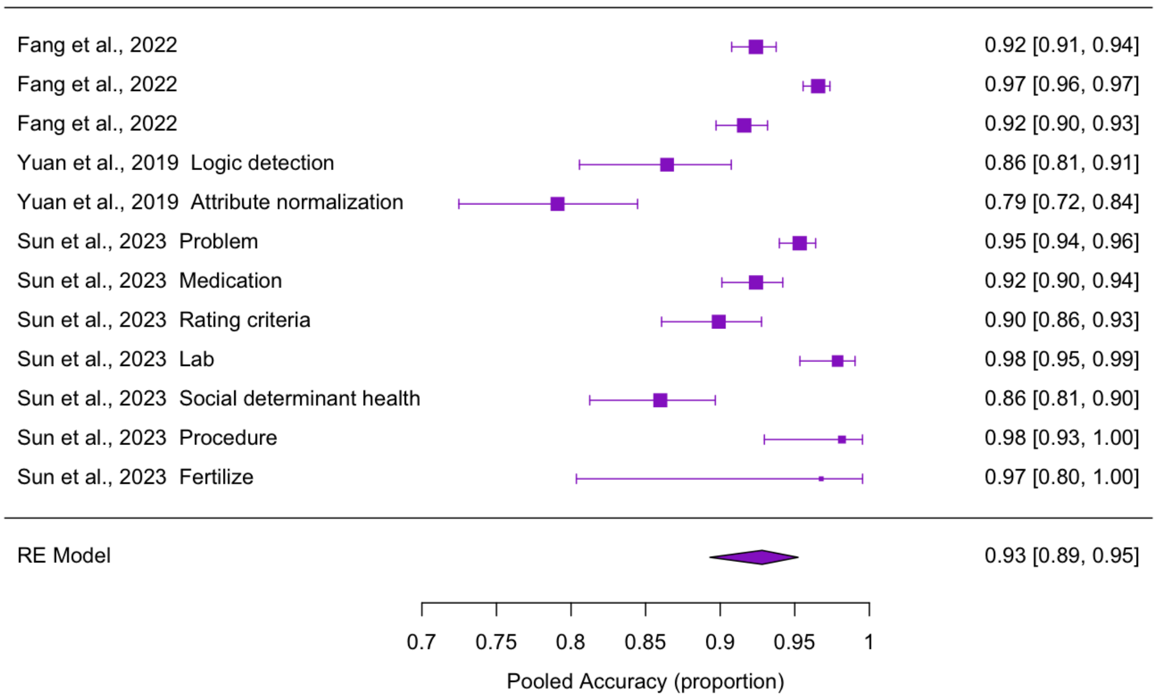

Figure 10. F-1 Score Meta-analysis for Papers Using Machine Learning to Identify Eligibility Criteria

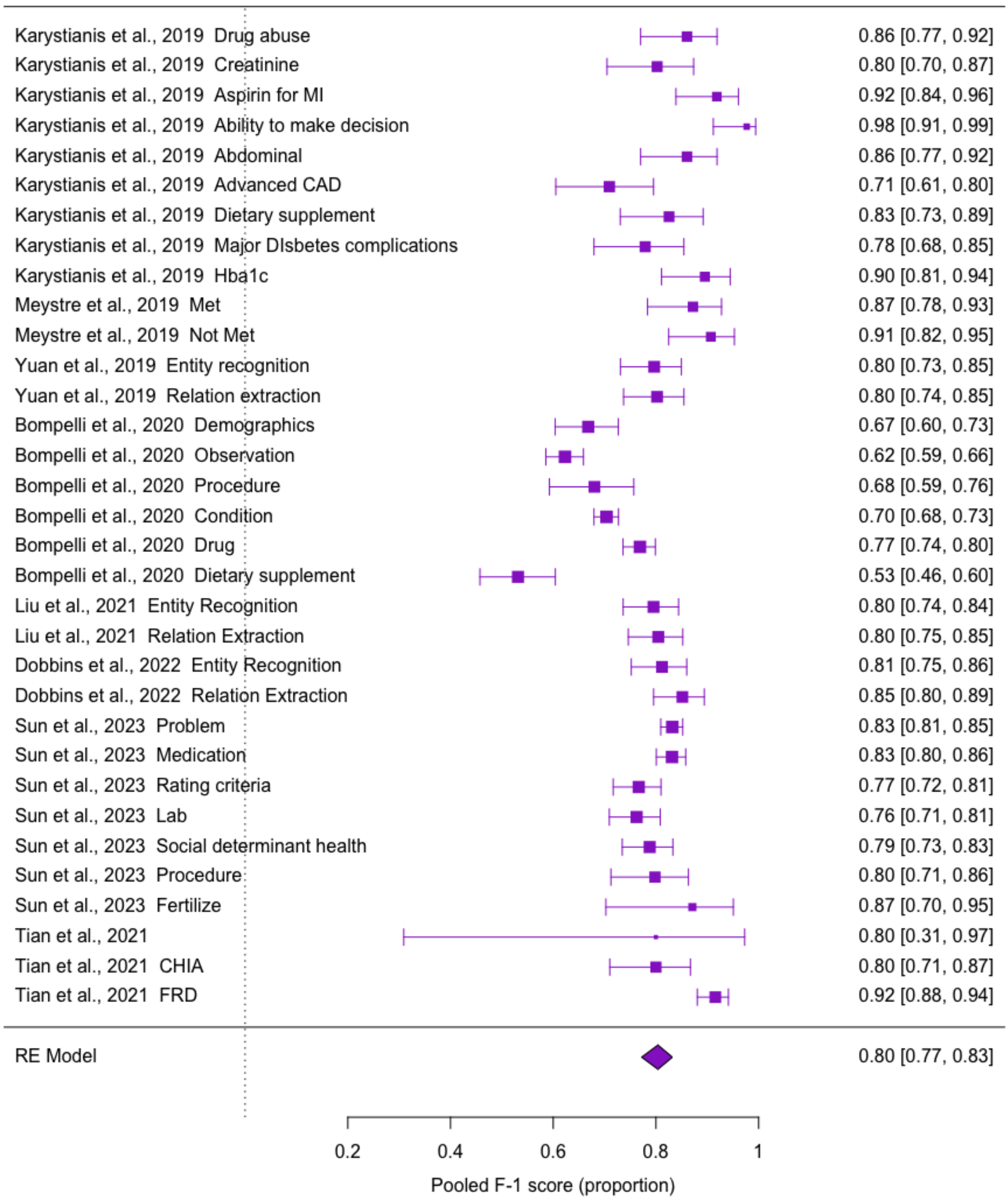

Figure 11. Sensitivity of Meta-analysis for Papers Using Machine Learning to Classify Eligibility Criteria

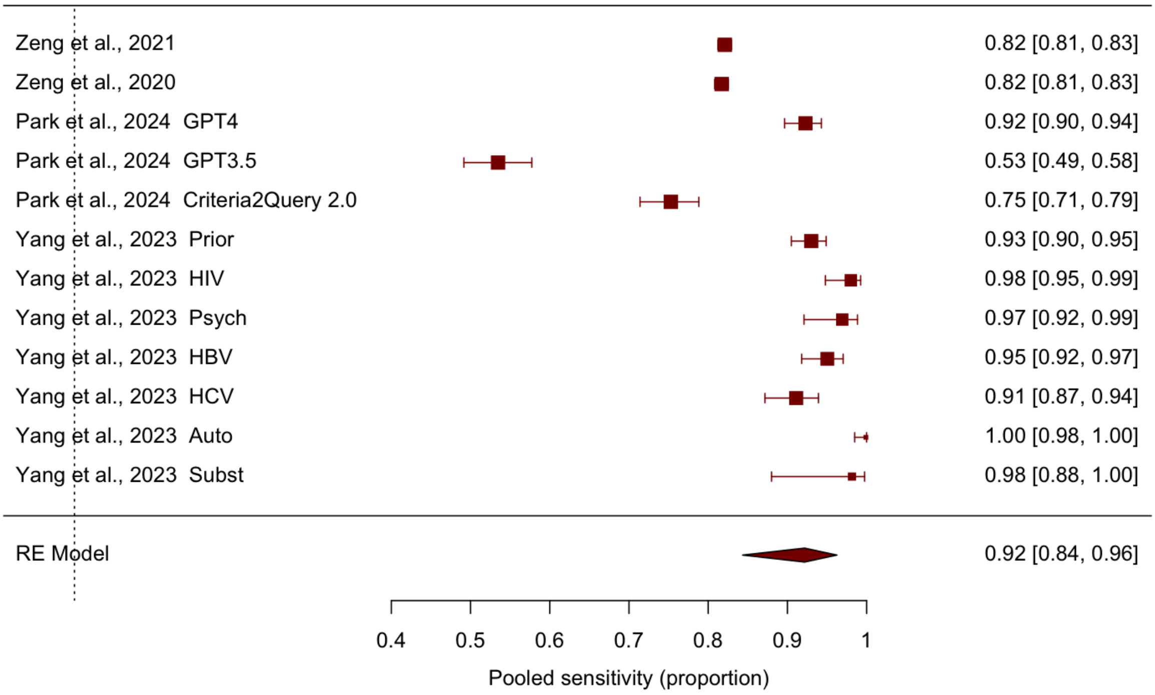

Figure 12. Precision Meta-analysis for Papers Using Machine Learning to Classify Eligibility Criteria

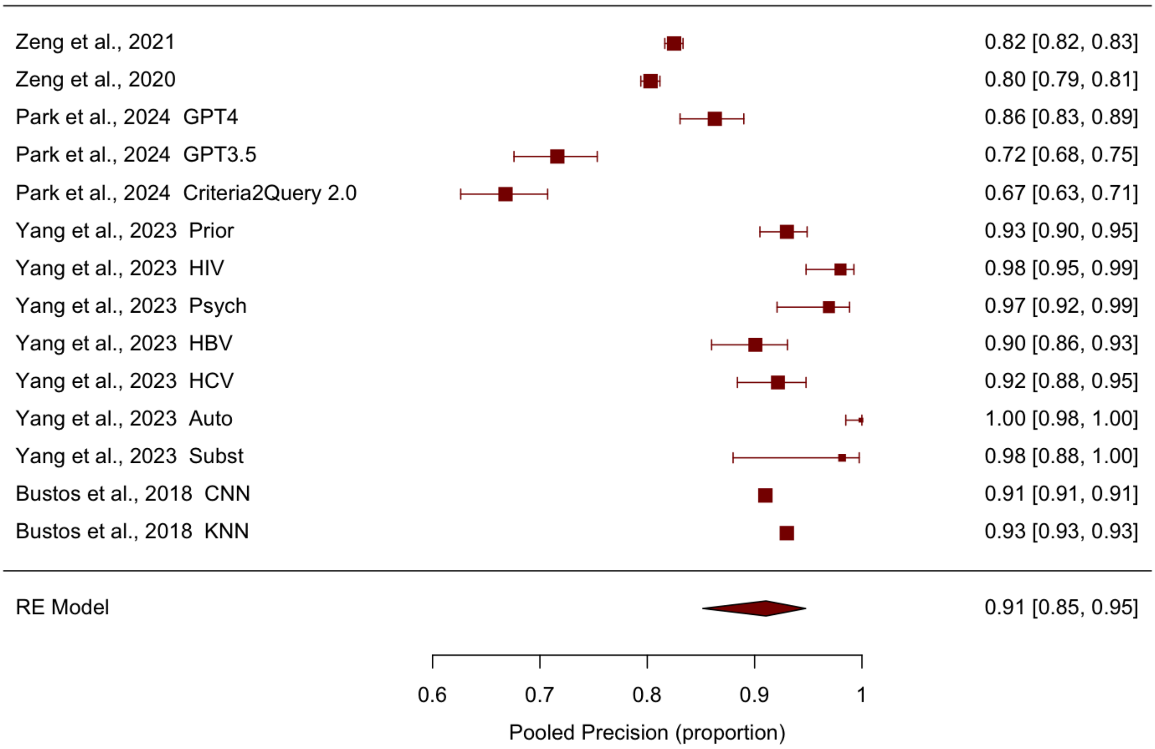

Figure 13. Accuracy Meta-analysis for Papers Using Machine Learning to Classify Eligibility Criteria

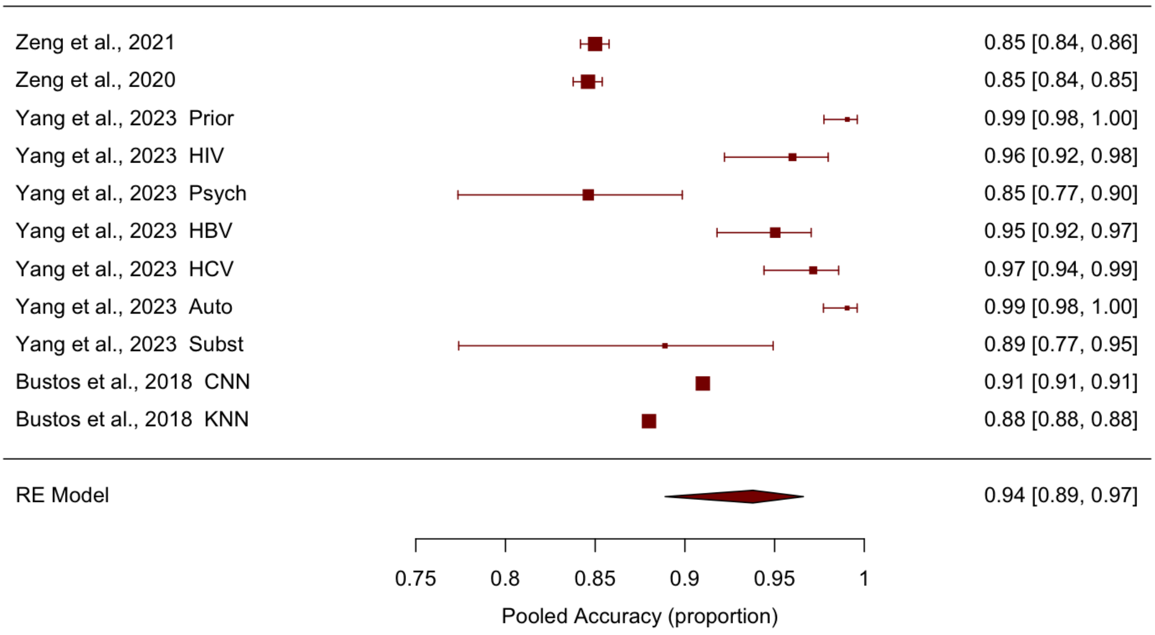

Figure 14. F-1 Score Meta-analysis for Papers Using Machine Learning to Classify Eligibility Criteria

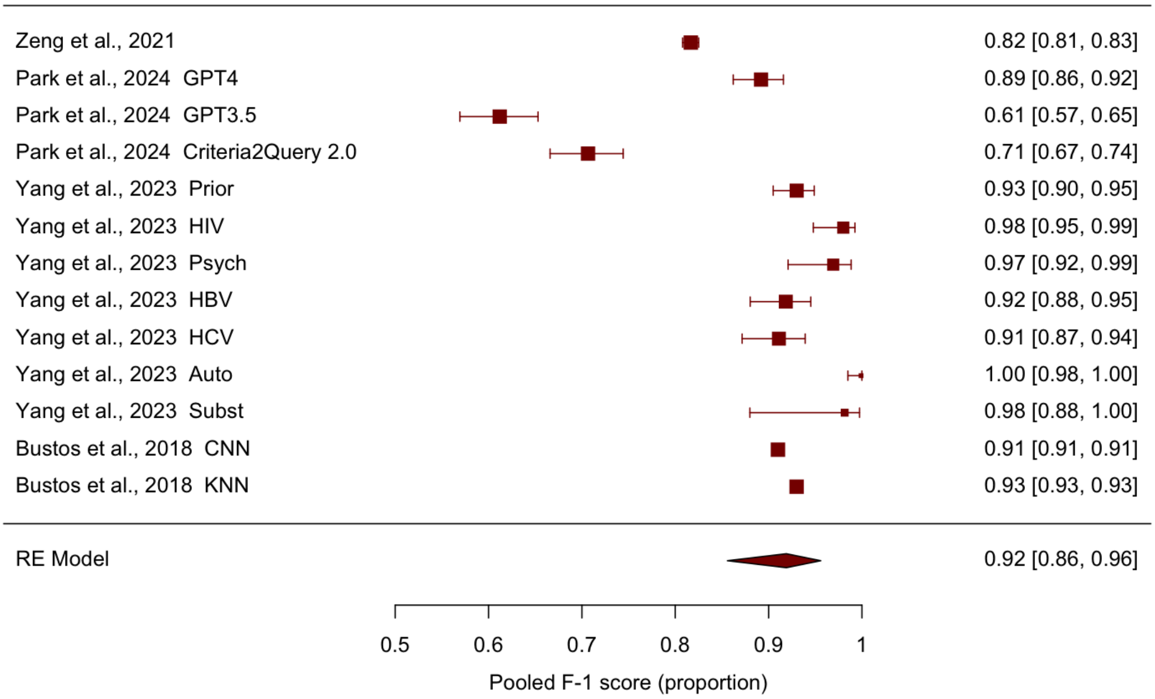

Figure 15. Sensitivity Meta-analysis for Papers Using Machine Learning to Identify Petients/ Cohort

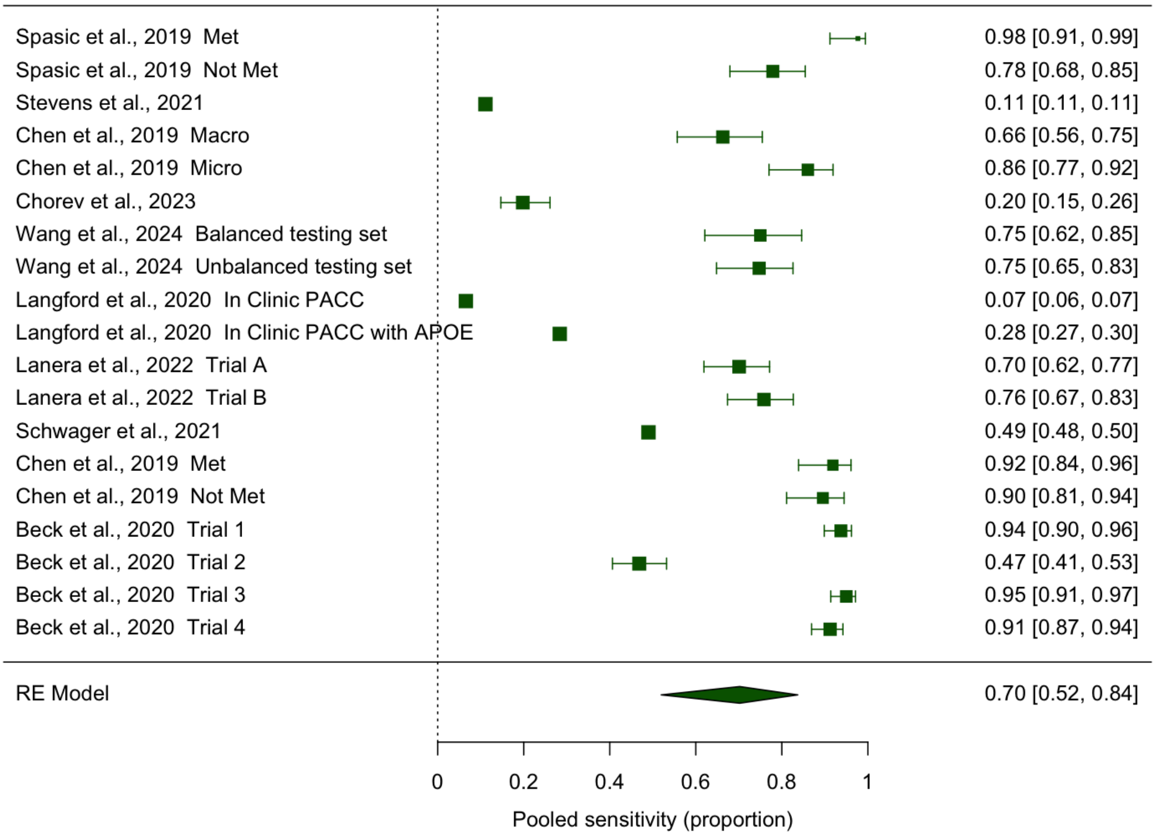

Figure 16. Specificity Meta-analysis for Papers Using Machine Learning to Identify Petients/ Cohort

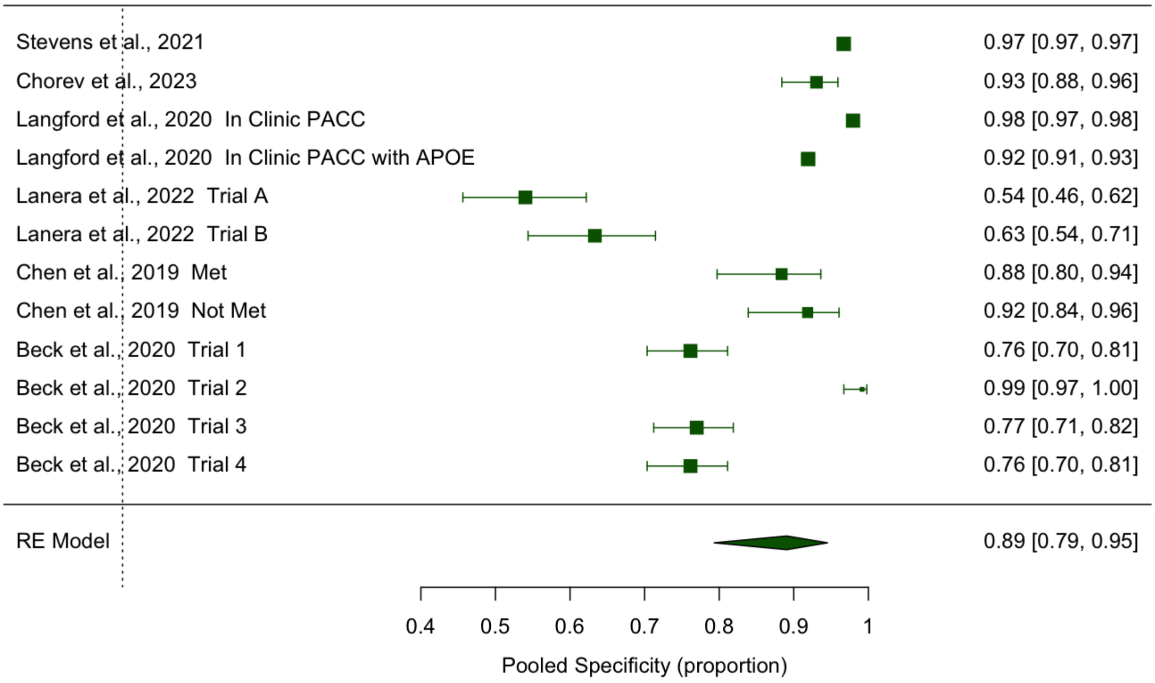

Figure 17. Precision Meta-analysis for Papers Using Machine Learning to Identify Petients/ Cohort

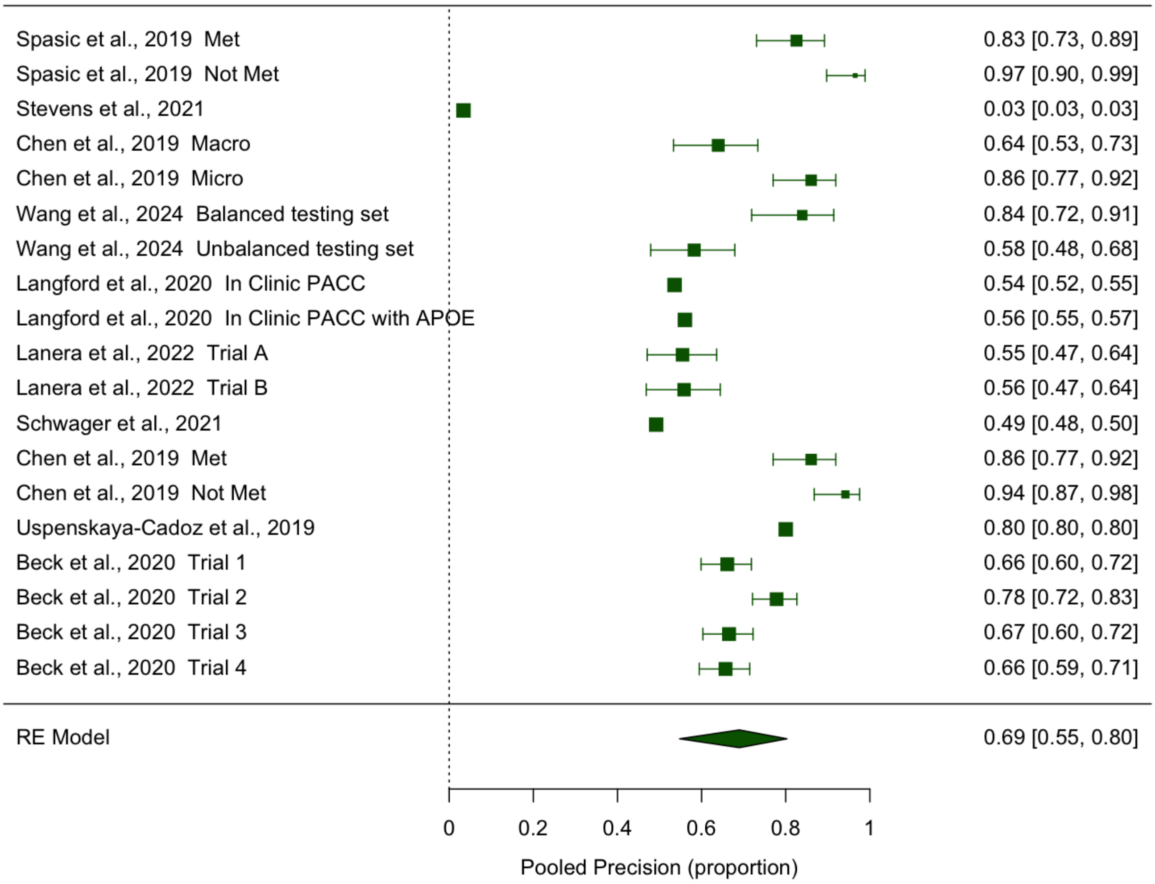

Figure 18. Accuracy Meta-analysis for Papers Using Machine Learning to Identify Petients/ Cohort

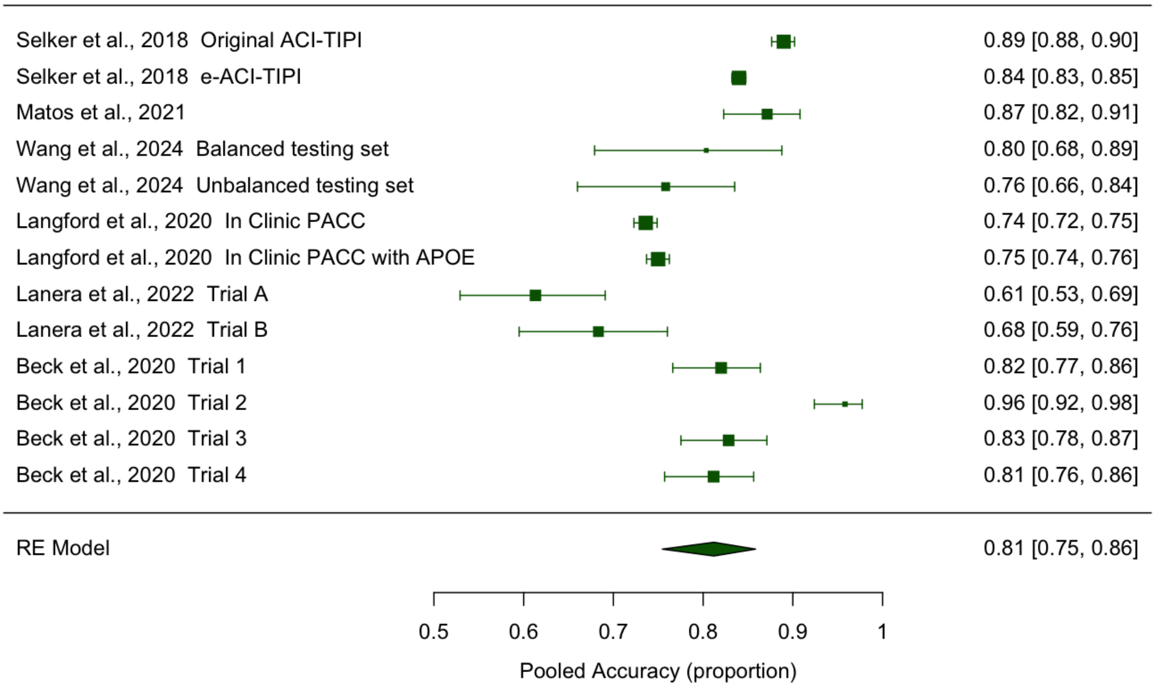

Figure 19. AUC Meta-analysis for Papers Using Machine Learning to Identify Petients/ Cohort

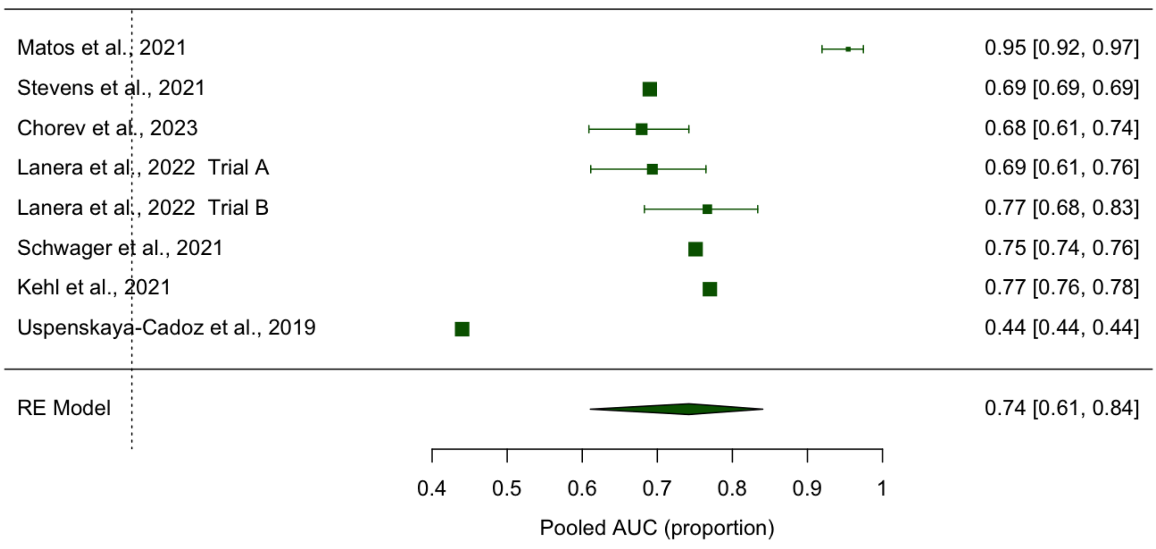

Figure 20. F-1 Score Meta-analysis for Papers Using Machine Learning to Identify Petients/ Cohort

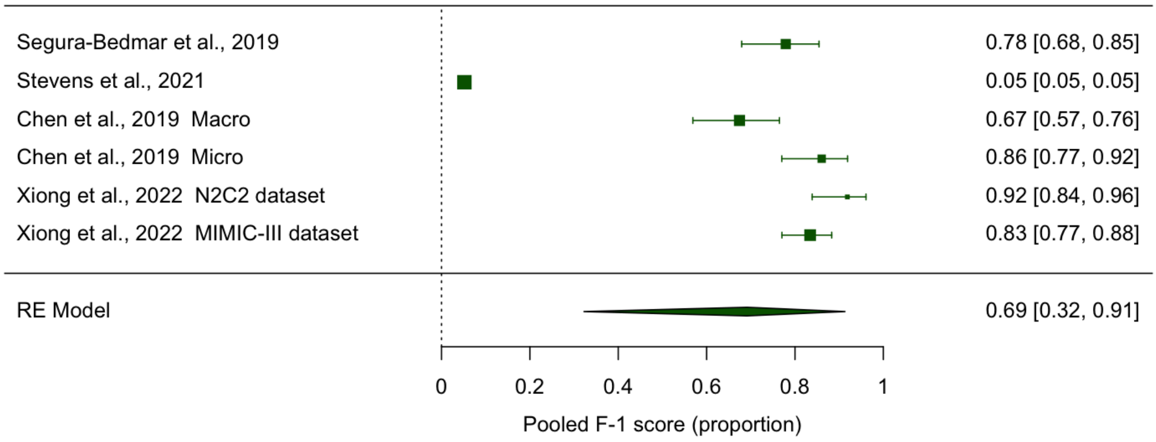

Appendix D: Others

Summary of Reported Matrices

Screen patients

- sensitivity: 0.400 (8/20)
- specificity: 0.150 (3/20)
- accuracy: 0.100 (2/20)
- precision (ppv): 0.200 (4/20)
- f-1: 0.300 (6/20)
- auc: 0.300 (6/20)

Identify patients

- sensitivity: 0.333 (10/30)
- specificity: 0.200 (6/30)
- accuracy: 0.200 (6/30)
- precision (ppv): 0.333 (10/30)
- f-1: 0.133 (4/30)
- auc: 0.233 (7/30)

Identify eligibility criteria

- sensitivity: 0.571 (8/14)
- specificity: 0 (0/14)
- accuracy: 0.214 (3/14)
- precision (ppv): 0.571 (8/14)
- f-1: 0.571 (8/14)
- auc: 0 (0/14)

Classify eligibility criteria

- sensitivity: 0.500 (3/6)
- specificity: 0 (0/6)
- accuracy: 0.500 (3/6)
- precision (ppv): 0.667 (4/6)
- f-1: 0.667 (4/6)
- auc: 0 (0/6)

Figure 3 Details

Country

	Country	n	prop	percent
1	United States	74	0.622	62.18%
2	United Kingdom	9	0.0756	7.56%
3	China	8	0.0672	6.72%
4	France	5	0.0420	4.20%
5	Australia	3	0.0252	2.52%
6	Germany	3	0.0252	2.52%
7	Italy	3	0.0252	2.52%

		n	prop	percent
8	Spain	3	0.0252	2.52%
9	Austria	2	0.0168	1.68%
10	Taiwan	2	0.0168	1.68%
11	Colombia	1	0.00840	0.84%
12	Lebanon	1	0.00840	0.84%
13	Netherlands	1	0.00840	0.84%
14	Nigeria	1	0.00840	0.84%
15	Romania	1	0.00840	0.84%
16	Switzerland & USA	1	0.00840	0.84%
17	USA & China	1	0.00840	0.84%

<u>Addressing Bias/Disparities</u>

	Bias_Addressed_YN	n	prop	percent
1	No	104	0.860	85.95%
2	Yes	17	0.140	14.05%

<u>Publication Year</u>

	Year	n	prop	percent
1	2021	27	0.225	22.50%
2	2023	22	0.183	18.33%
3	2024	19	0.158	15.83%
4	2019	18	0.15	15.00%
5	2020	15	0.125	12.50%
6	2022	11	0.0917	9.17%
7	2018	8	0.0667	6.67%

<u>AI Approaches</u>

	AI_Approaches	n	prop	percent
1	Machine learning AI	87	0.725	72.50%
2	Classification/ data management	17	0.142	14.17%
3	Message reminder/ chatbot/ offering information	10	0.0833	8.33%
4	Intervention for better engagement	6	0.05	5.00%

<u>Timing (Retrospective vs. Real-Time Studies)</u>

	Timing_clean	n	prop	percent
1	Retrospective	88	0.772	77.19%
2	Real time	26	0.228	22.81%

<u>Implementation (Theoretical vs. Actual)</u>

	Implementation	n	prop	percent
1	Theoretical	63	0.548	54.78%
2	Actual	52	0.452	45.22%

Screen patients I workflow_category (6).xlsx I Sensitivity

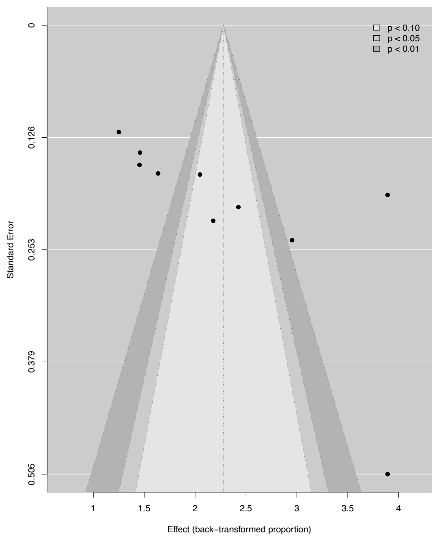

Screen patients I workflow_category (6).xlsx I F−1 score

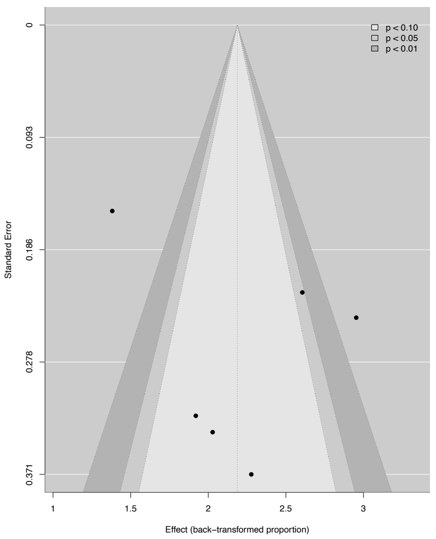

Screen patients I workflow_category (6).xlsx I Precision

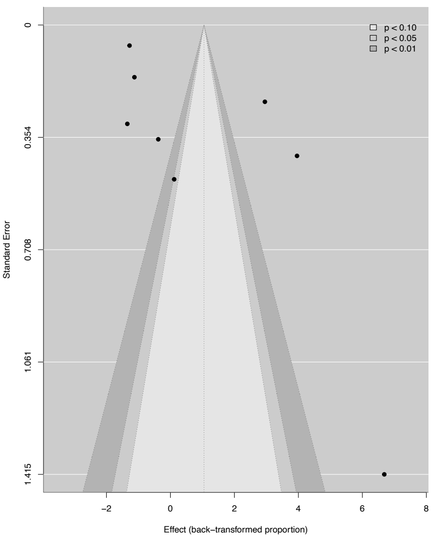

Screen patients I workflow_category (6).xlsx I Accuracy

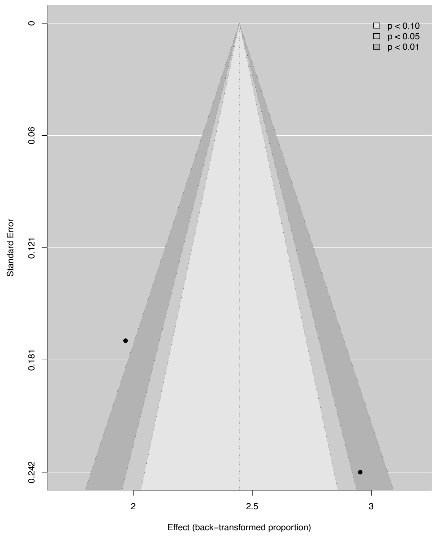

Screen patients I workflow_category (6).xlsx I Specificity

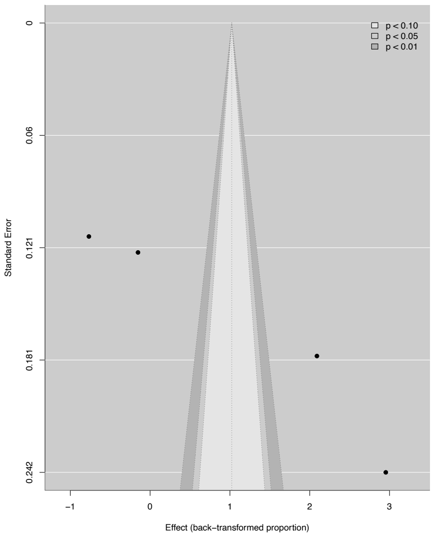

Screen patients I workflow_category (6).xlsx I AUC

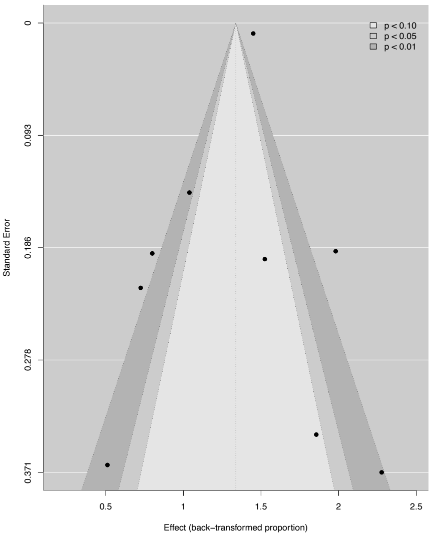

Identify patients I workflow_category (6).xlsx I Sensitivity

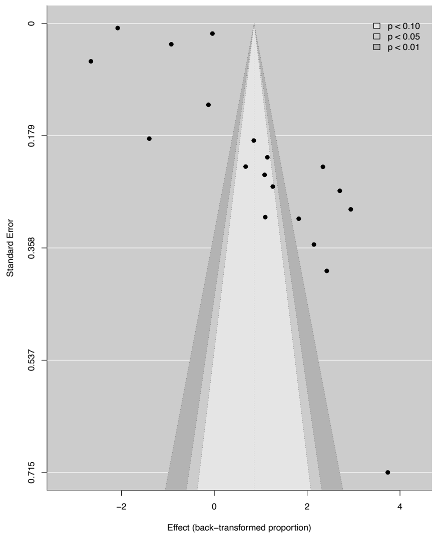

Identify patients I workflow_category (6).xlsx I F−1 score

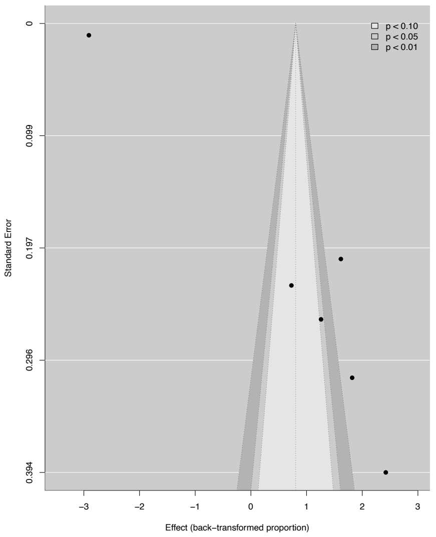

Identify patients I workflow_category (6).xlsx I Precision

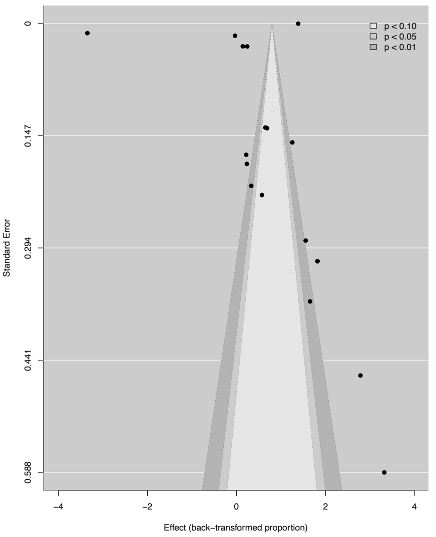

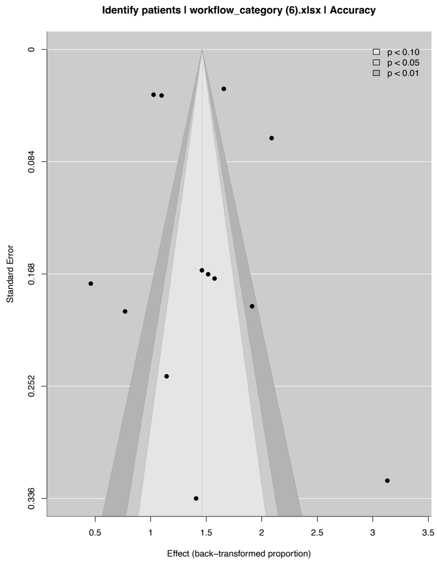
Identify patients | workflow_category (6).xlsx | Accuracy
p < 0.10
p < 0.05
p < 0.01
Standard Error
0
0.084
0.168
0.252
0.336
0.5
1
1.5
2
2.5
3
3.5
Effect (back−transformed proportion)

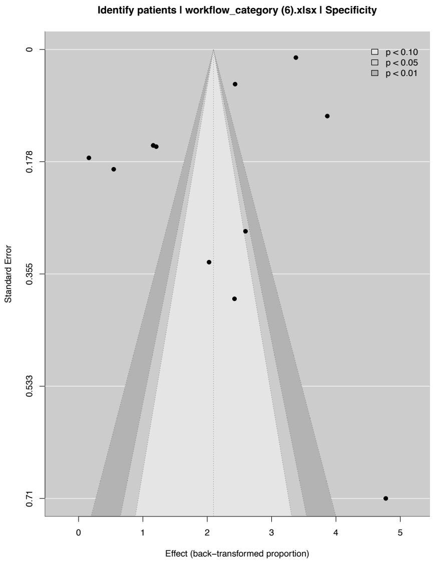
Identify patients | workflow_category (6).xlsx | Specificity
p < 0.10
p < 0.05
p < 0.01
Standard Error
0
0.178
0.355
0.533
0.71
0
1
2
3
4
5
Effect (back−transformed proportion)

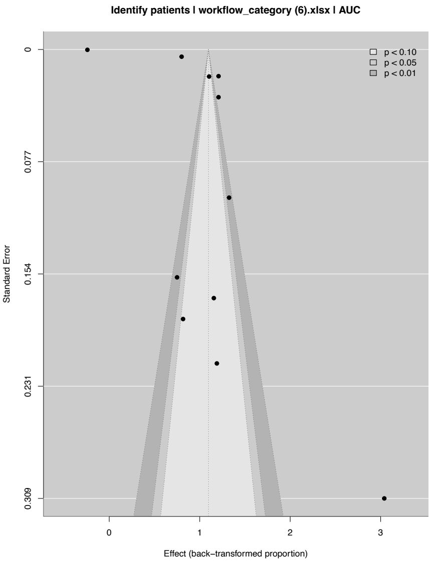
Identify patients | workflow_category (6).xlsx | AUC
p < 0.10
p < 0.05
p < 0.01
Standard Error
0
0.077
0.154
0.231
0.309
0
1
2
3
Effect (back−transformed proportion)

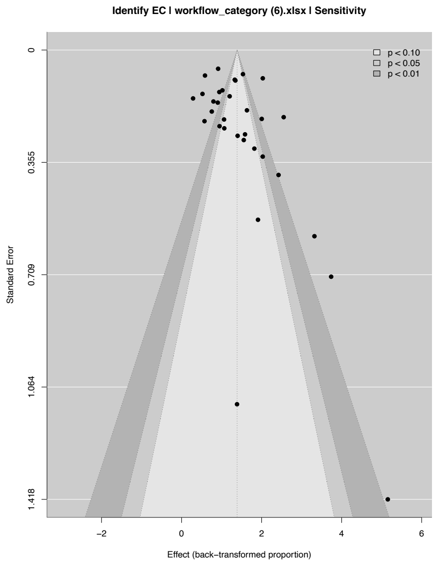
Identify EC | workflow_category (6).xlsx | Sensitivity
p < 0.10
p < 0.05
p < 0.01
Standard Error
0
0.355
0.709
1.064
1.418
−2
0
2
4
6
Effect (back−transformed proportion)

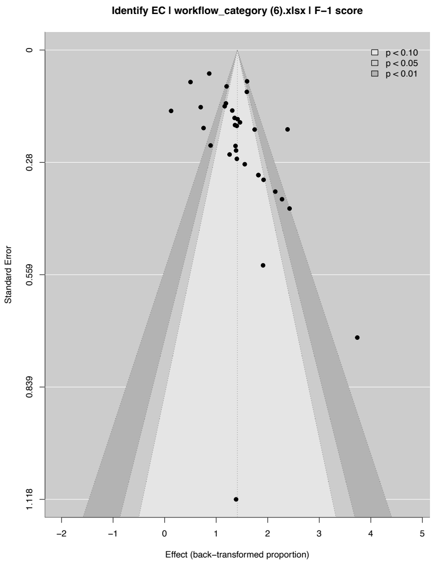
Identify EC | workflow_category (6).xlsx | F−1 score
p < 0.10
p < 0.05
p < 0.01
Standard Error
0
0.28
0.559
0.839
1.118
−2
−1
0
1
2
3
4
5
Effect (back−transformed proportion)

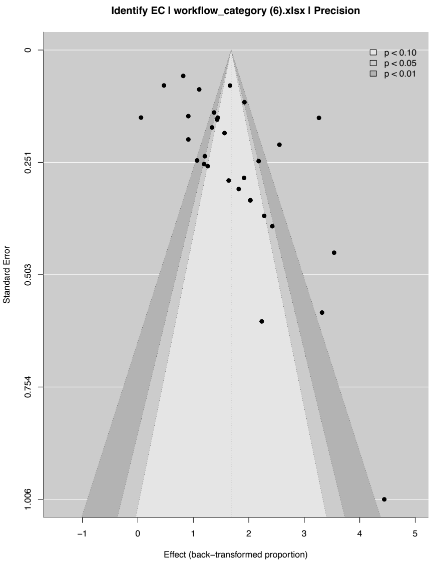
Identify EC | workflow_category (6).xlsx | Precision
p < 0.10
p < 0.05
p < 0.01
Standard Error
0
0.251
0.503
0.754
1.006
−1
0
1
2
3
4
5
Effect (back−transformed proportion)

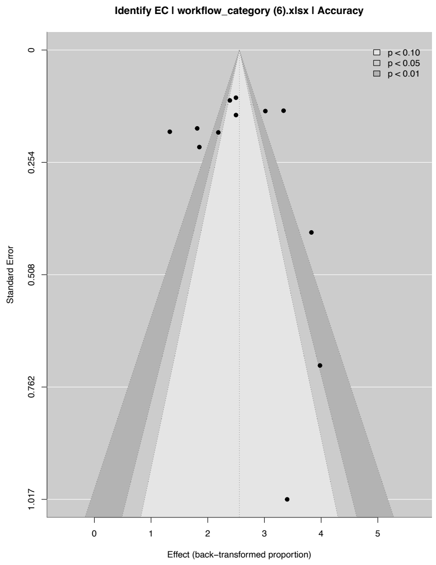
Identify EC | workflow_category (6).xlsx | Accuracy
p < 0.10
p < 0.05
p < 0.01
Standard Error
0
0.254
0.508
0.762
1.017
0
1
2
3
4
5
Effect (back−transformed proportion)

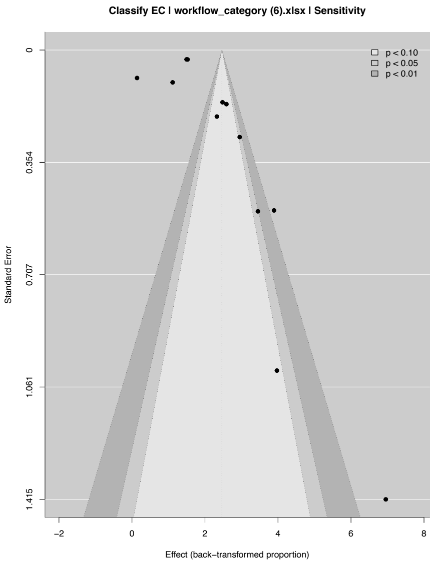
Classify EC | workflow_category (6).xlsx | Sensitivity
p < 0.10
p < 0.05
p < 0.01
Standard Error
0
0.354
0.707
1.061
1.415
−2
0
2
4
6
8
Effect (back−transformed proportion)

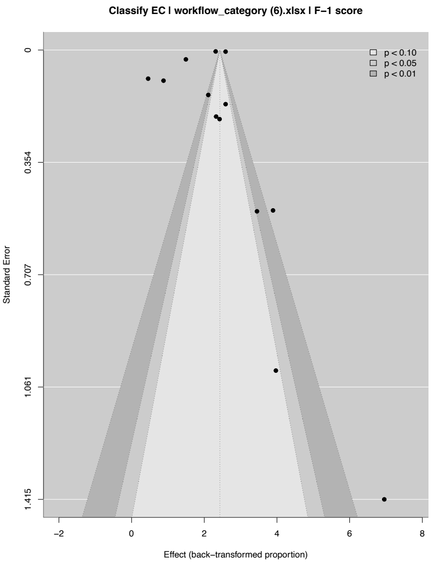
Classify EC | workflow_category (6).xlsx | F−1 score
p < 0.10
p < 0.05
p < 0.01
Standard Error
0
0.354
0.707
1.061
1.415
−2
0
2
4
6
8
Effect (back−transformed proportion)

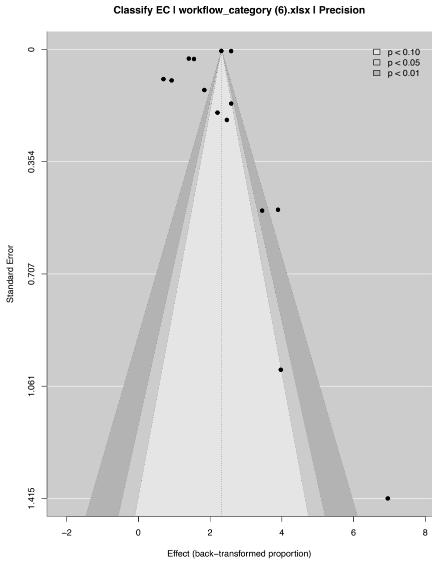
Classify EC | workflow_category (6).xlsx | Precision
p < 0.10
p < 0.05
p < 0.01
Standard Error
0
0.354
0.707
1.061
1.415
−2
0
2
4
6
8
Effect (back−transformed proportion)

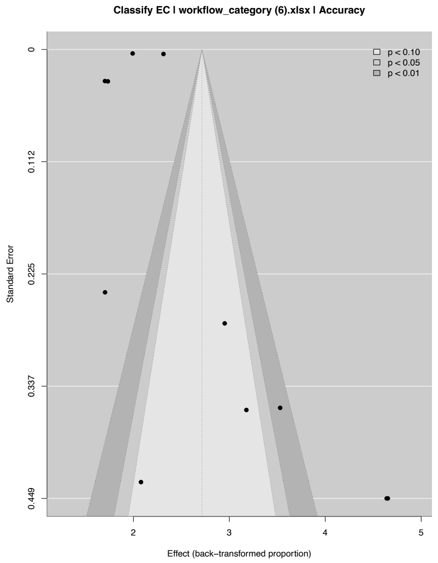
Classify EC | workflow_category (6).xlsx | Accuracy
p < 0.10
p < 0.05
p < 0.01
Standard Error
0
0.112
0.225
0.337
0.449
2
3
4
5
Effect (back−transformed proportion)
